# Molecular Mechanisms of Treatment Resistance in Glioblastoma

**DOI:** 10.3390/ijms22010351

**Published:** 2020-12-31

**Authors:** Alexander Ou, W. K. Alfred Yung, Nazanin Majd

**Affiliations:** Department of Neuro-Oncology, The University of Texas MD Anderson Cancer Center, 1515 Holcombe Blvd., Unit 431, Houston, TX 77030, USA; aou@mdanderson.org

**Keywords:** glioblastoma, heterogeneity, chemoresistance, radioresistance, immunotherapy, targeted therapy

## Abstract

Glioblastoma is the most common malignant primary brain tumor in adults and is almost invariably fatal. Despite our growing understanding of the various mechanisms underlying treatment failure, the standard-of-care therapy has not changed over the last two decades, signifying a great unmet need. The challenges of treating glioblastoma are many and include inadequate drug or agent delivery across the blood–brain barrier, abundant intra- and intertumoral heterogeneity, redundant signaling pathways, and an immunosuppressive microenvironment. Here, we review the innate and adaptive molecular mechanisms underlying glioblastoma’s treatment resistance, emphasizing the intrinsic challenges therapeutic interventions must overcome—namely, the blood–brain barrier, tumoral heterogeneity, and microenvironment—and the mechanisms of resistance to conventional treatments, targeted therapy, and immunotherapy.

## 1. Introduction

Glioblastoma (GBM) is the most common malignant primary brain tumor in adults, and despite standard-of-care multimodality therapy, including maximal safe resection, radiotherapy, and chemotherapy, the prognosis remains almost universally fatal with a mean overall survival of 14 to 20 months [1]. Since the 2005 pivotal phase III trial by Stupp et al. [1], which established the role of concurrent chemoradiation with temozolomide followed by adjuvant temozolomide for patients with newly diagnosed glioblastoma, no chemotherapies investigated in late-phase clinical trials have significantly improved upon this foundational approach. The U.S. Food and Drug Administration (FDA) has approved the anti-vascular endothelial growth factor (VEGF) antibody bevacizumab for treatment of recurrent glioblastoma on the basis of two phase II studies showing a progression-free survival benefit. However, two phase III clinical trials evaluating its role in the treatment of newly diagnosed disease did not demonstrate an overall survival benefit when bevacizumab was added to standard therapy [2,3,4]. The FDA also has approved tumor-treating fields therapy (TTF), which consists of low-intensity, alternating electric fields applied to the scalp for most of the day, for use in recurrent (2011) and newly diagnosed (2015) glioblastoma, although widespread adoption of TTF has been limited by methodological concerns about the generalizability of the data from prior studies of it [5]. Thus, there are currently no effective therapies for glioblastoma. In this review, we discuss the innate mechanisms of treatment resistance common to all glioblastomas before characterizing the various mechanisms of resistance to conventional treatments, targeted therapies, and immunotherapy.

## 2. General Mechanisms of Treatment Resistance

### 2.1. The Blood–Brain and Blood–Brain–Tumor Barriers

**Figure 1:**
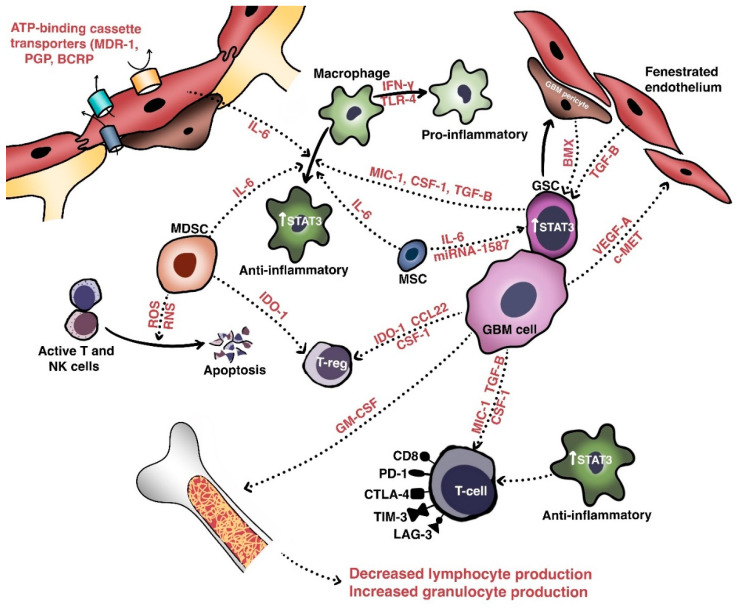
Anatomic, cellular and molecular basis of treatment resistance in GBM. The tumoral microenvironment of GBM consists of the blood–brain barrier and a number of important populations of cells: glioma cells, glioma stem cells, and various important immune cells including tumor-associated macrophages, tumor-infiltrating lymphocytes, and myeloid-derived suppressor cells. Crosstalk between these cells occurs via involves various cytokines and growth factors, the net effect of which results in a stemness-promoting, proliferative, angiogenic, and immunosuppressive milieu.

The initial obstacle that therapies against malignant gliomas must overcome is the blood-brain barrier (BBB), a non-fenestrated physical barrier comprised of specialized capillary endothelial cells interconnected by multi-protein tight junctions consisting of claudins (especially claudin-1, -3, and -5), occludins, and junctional adhesion molecules [6]. Closely associated with these endothelial cells by virtue of a shared basal lamina are complexes of astrocytic endfeet, pericytes, and intermittent ends of neurons, which collectively constitute the neurovascular unit responsible for maintaining biochemical and physical homeostasis in the normal brain [7]. The BBB permits only small (<500 Da and <400 nm) and lipophilic molecules to passively diffuse across; other molecules cross the BBB via pinocytosis, receptor- or carrier-mediated transcytosis, and solute-carrier-protein mechanisms [8]. The integrity of the BBB and homeostatic equilibrium are further bolstered by ATP-binding cassette transporters, such as multidrug resistance-1 (MDR1), P-glycoprotein, breast cancer resistance protein, and numerous other drug resistance proteins that are expressed on the luminal and abluminal sides of vessel walls (Figure 1). These transporters actively mediate the efflux of xenobiotics such as cytotoxic or targeted therapeutic agents out of the brain parenchyma [9,10,11]. Attempts to modulate these efflux pumps have largely been unsuccessful [12,13].

In glioblastoma and other high-grade intracranial neoplasms, the BBB is heterogeneously disrupted to form the blood–tumor–brain barrier, which is characterized by abnormal pericyte distribution, reduced tight junctions, the loss of astrocytic endfeet, and increased permeability to circulating immune cells [14,15]. This heterogeneity of tumoral vasculature creates regional niches of variable permeability to blood, oxygen, nutrients, and drugs. Recent work has further extended our understanding of a glioblastoma tumor’s centrally necrotic, hypoxic core, and less-hypoxic periphery [16]. Indeed, hypoxic glioblastoma cells secrete VEGF-A via exosomes to promote the proliferation of endothelial cells with downregulated expression of key junctional proteins such as claudin-5 and occludin [17,18].

Glioma stem cells (GSCs), which are pluripotent, slowly dividing, and therapy-resistant cells residing in the perivascular hypoxic niches of the brain, have been recognized for their importance in resisting cytotoxic therapies [19,20]. GSCs are not only intrinsically resistant to therapy but also exert substantial effects on neighboring cells within the microenvironment to maintain their populations [21]. In particular, glioblastoma pericytes derive from GSCs via trans-differentiation and contribute to the integrity of the BBB via the overexpression of proteins such as bone marrow and X-linked (BMX) non-receptor tyrosine kinase (Figure 1), which activate signaling through signal transducer and activator or transcription 3 (STAT3) to maintain the self-renewal capability of the GSCs occupying perivascular niches [14,22]. Indeed, in an orthotopic xenograft glioblastoma model, Zhou et al. [23] found that pericyte coverage not only correlated with the prognosis of patients with glioblastoma but also that inhibition of BMX with ibrutinib selectively disrupted the permeability of the blood–brain–tumor barrier and enhanced delivery of chemotherapy (e.g., etoposide) that ordinarily penetrates the BBB poorly, thus prolonging mouse survival [24].

The two most important signaling pathways involved in the formation of the BBB and the regulation of its integrity are the Wingless-related integration site (Wnt) and Sonic hedgehog (Shh) pathways. During normal embryonic development and in adulthood, Shh secreted by astrocytes binds to the patched-1 (Ptch-1) protein on endothelial cells or pericytes to activate the smoothened (Smo) protein. This leads to downstream transcriptional activation of genes bound by the Gli family of transcription factors, such as SOX-18 and TAL-1, which increase transendothelial resistance and decrease permeability by enhancing claudin expression [25,26,27]. Shh signaling also contributes to the central nervous system’s immune privilege by decreasing the expression of the intercellular adhesion molecule ICAM-1 and the secretion of chemokines such as CXCL8/interleukin (IL)-9 and CCL2/MCP-1 by endothelial cells. Similarly, Wnt/β-catenin signaling in endothelial cells contributes to the regulation of the BBB. The endothelial G-protein coupled receptor Gpr124 is one such crucial coactivator of Wnt7a and Wnt7b-stimulated canonical signaling via the binding of Frizzled receptor and Lrp coreceptor. Gpr124 upregulates claudin-5 expression, decreases platelet-derived growth factor receptor (PDGFR)-B expression, and increases pericyte coverage [28]. Recently, Griveau et al. [29] extended the understanding of glioma cellular phenotypes vis-à-vis the tumoral microenvironment, demonstrating in a mouse model that Olig2+/Wnt7+ glioma cells—analogous to the oligodendrocytes comprising the leading edge of glioblastoma tumors—invaded the brain parenchyma via co-option of blood vessels by single-cells, while Olig2-/Wnt7- glioma cells—analogous to proneural glioblastoma cells—proliferated in the perivascular niche and expressed abundant VEGF-C and VEGFR-1/2/3 to form dense tumor collections with leaky vasculature. Importantly, anti-angiogenic therapy (i.e., VEGF inhibition) led to selective enrichment of the Olig2+/Wnt7+ cells, indicating a mechanism through which glioblastoma cells may ultimately overcome prolonged anti-angiogenic therapy.

Strategies to breach the BBB and improve drug delivery have therefore focused on mechanical disruption (i.e., osmotic disruption) and invasive local delivery (e.g., convection-enhanced delivery), and these strategies have been limited by either unacceptable toxicity or inefficacy [30,31,32]. Focused ultrasound is a relatively new modality which transiently renders the BBB permeable to allow for improved drug delivery with a more favorable adverse effect profile, and clinical study is underway [33]. Continued efforts to improve drug delivery via nanoparticle- or peptide-based drug-carrying methods are ongoing, and further study of pharmacological inhibition of Wnt/Shh signaling and pericyte function is warranted [34,35].

### 2.2. Intra- and Intertumoral Heterogeneity

Perhaps the most important and challenging barrier to establishing effective treatments for glioblastoma is tumoral heterogeneity, which encompasses a vast spectrum of molecular, genetic, cellular, temporal, spatial, and evolutionary diversity and prevents the use of any single universal therapeutic approach. The Cancer Genome Atlas (TCGA) Research Network originally used an extensive characterization of the genomic alterations in glioblastoma to identify three critical signaling pathways in the disease—p53, retinoblastoma (Rb), and receptor tyrosine kinase/Ras/PI3K—and subsequent groups have built upon these data to formulate classification schemes with prognostic importance. Verhaak et al. [36,37] used factor analysis and consensus clustering of data from TCGA to define 4 glioblastoma subtypes on transcriptional grounds: classical, mesenchymal, proneural, and neural. The classical subtype is characterized by the gain of chromosome 7 and loss of chromosome 10, epidermal growth factor receptor (EGFR) amplification, and cyclin dependent kinase inhibitor 2A (CDKN2A) homozygous deletion with high-level upregulation of Notch (NOTCH1, NOTCH3, JAG1, LFNG)- and Shh (SMO, GAS1, GLI2)-related signaling with downregulation of mitogen-activated protein kinase (MAPK) and pro-apoptotic proteins such as cleaved caspase 7 and 9, Bid, and Bak. The mesenchymal subtype is characterized by mutations in NF1, phosphatase, and tensin-homolog protein (PTEN), and the nuclear factor κ-light chain-enhancer of activated B cells (NF-κB) signaling pathway (e.g., TRADD, RELB, and TNFRSF1A) with increased MAPK and decreased mTOR signaling. The proneural subtype is characterized by IDH1 mutations, PDGFRA amplification, TP53 mutation, PI3K signaling, and high expression levels of oligodendrocytic developmental genes (e.g., *OLIG2*, *NKDX2-2*, and unique genomic hypermethylation-designated glioma-CpG island methylator phenotype or G-CIMP+) [38,39,40]. The genuine existence of the neural subtype is controversial, as subsequent studies by Wang et al. [41] and Gill et al. [42] have suggested that sampling of non-neoplastic cells at the infiltrative margins of the tumor account for the transcriptional profile was observed. Whole exome and transcriptional sequencing and proteomic profiling have further refined our understanding of the molecular subtypes. Various EGFR alterations (e.g., gene deletions or fusions) have also been detected, signifying the sheer genetic complexity of glioblastoma.

A model of cellular states and genetic diversity in glioblastoma recently postulated by Neftel et al. [43] integrated single-cell RNA sequencing and bulk genomic/transcriptomic and single-cell lineage tracing to demonstrate that sets of genes—designated “meta-modules”and encompassing mesenchymal, astrocytic, oligodendroglial, stem cell, and neural progenitor cell programs—recurred at high rates between tumors despite substantial intratumoral heterogeneity. Cellular populations isolated on the basis of these meta-modules generally expressed only 1 meta-module, and 15% of cells expressed 2, suggesting a hybrid subtype. Importantly, multiple cellular states were found to coexist within each tumor, each partially dictated by genetic mutations such as EGFR or PDGFRA.

It remains to be seen how this knowledge of the complex genomic, transcriptomic, epigenomic, and proteomic programs may be best applied to develop a therapeutic strategy to treat glioblastoma. However, it is clear from the information above that any approach must be individualized to some degree.

#### 2.2.1. Cellular Heterogeneity

In glioblastoma, heterogeneity at the single-cell level has long been inferred because of the presence of multiple transcriptional subtypes and subclones coexisting within the same tumor [44]. It is now understood that glioblastoma tumors consist of discrete populations of cells, each with a specific transcriptional signature consistent with a proneural, classical, or mesenchymal subtype. Tumors are further sustained by populations of GSCs expressing cell-surface markers (e.g., CD133, DDR1, and CD151) that divide slowly, share concordant genomic driver mutations (e.g., *TERT* promoter mutations, the gain of chromosome 7 and loss of chromosome 10), and are largely unaffected by therapies [45,46,47]. Multiple studies have established that these heterogeneous populations of GSCs are the primary source of intratumoral heterogeneity [46,48,49]. Single-cell transcriptional analysis has revealed well-defined subpopulations of GSCs, either expressing CD133 and stemness and neuron-related genes (e.g., *SOX11*, *PROM1*, *NES*, and *DDR1*) or CD151 and novel surface marker, stemness, and growth factor signaling genes (e.g., *CD44*, *FGF2*, and *PDGFRA*). In other words, transcriptional subtype heterogeneity originates from and is sustained by discrete stem cell populations that are intrinsically chemo- and radioresistant [50].

#### 2.2.2. Spatial Heterogeneity

Glioblastomas are also spatially heterogeneous owing to the niches created by hypoxia gradients. Indeed, on contrast-enhanced MRI, glioblastoma is characterized by heterogeneous enhancement and central necrosis, implying a highly vascular yet hypoxic core with a relatively oxygen-rich periphery. Single-cell RNA sequencing of core tumor cells has shown markedly upregulated expression of hypoxia genes (e.g., *PGK1*, *VEGF*-*A SPP1*, *HIF1A*, and *CA9*) and peritumoral infiltrative cells largely enriched for genes involved in cell–cell adhesion (e.g., *ECM2*, *ANGPT1*, *TSPAN7*), size regulation (*ATP1A2*), and survival (*FGFR3*, *LMO3*) [16]. Importantly, tumor-associated myeloid cells differed between the 2 milieus, with the macrophages accounting for most of the immune cells in the core and microglia accounting for those in the periphery. These myeloid cells were associated with upregulation of pro-inflammatory markers in the periphery (e.g., IL1A/B) and more anti-inflammatory and pro-angiogenic markers (e.g., IL1RN, VEGF-A, TGFBI) in the core. Of note, large inter-tumor variation in the expression of immune-checkpoint-receptor ligands was also observed, indicating that not all patients will respond to checkpoint inhibition strategies.

There is evidence that glioblastoma spatial heterogeneity is partly dictated by the transcriptional subtype. Mesenchymal glioblastomas, for instance, have large numbers of tumor-associated macrophages of the immunosuppressive phenotype—i.e., those expressing integrin alpha M (ITGAM) or allograft inflammatory factor 1 (AIF1)—with small and more uniformly-distributed vasculature. Proneural glioblastomas have disorganized vasculature [51]. Classical tumors, on the other hand, frequently express activated dendritic cell signatures [41]. Understanding the tumoral microenvironment is critical to designing rational therapeutic strategies.

Although glioblastoma’s complex heterogeneity has historically been a major obstacle in the design of adequate and faithful preclinical models of the disease, recent work may help researchers overcome this obstacle. Jacob et al. [52] generated glioblastoma organoids without mechanical or enzymatic dissociation and in optimized medium for maintenance without added growth factors or extracellular matrices. This allowed for the preservation of the local cytoarchitecture and intercellular interactions present in the original tumor. Importantly, these organoids not only maintained the characteristic cellular, transcriptional, and molecular signatures of their parental tumors—including those of nonneoplastic cells such as macrophages and microglia—but even developed hypoxia gradients. This substantial technical advance will allow investigators to replicate individualized tumoral microenvironments.

#### 2.2.3. Heterogeneity between Primary and Recurrent Tumors

Acknowledging the limitations of a single time-point analysis, multiple groups have explored the complex genomic and epigenomic changes in glioblastoma by analyzing both primary and recurrent tumors.

Kim et al. [53] performed whole-genome and multisector exomic sequencing of untreated and firstly-recurrent glioblastoma and found that TP53 and PIK3CA/PIK3R1 mutations were almost entirely clonal and that receptor tyrosine kinase genes such as *EGFR*, *PDGFRA*, and *AKT* were just as likely to be subclonal as clonal. They further determined that p53 pathway deregulation and IDH1 mutation were associated with increased subclonal mutations and that most primary glioblastoma mutations were detectable in relapsed tumors. Signaling pathways involving FGF, PDGFR, and EGFR were also altered at the time of recurrence [54]. Interestingly, copy number alterations and single-nucleotide variants in genes such as TP53, EGFR, and CDKN2A were often not present in recurrent samples, suggesting that selection pressures exist within tumors and result in convergent evolutionary events. Kim et al. also discovered 2 patterns of disease relapse whereby recurrent tumors (1) no longer shared clonal mutations or (2) shared a high degree of clonal-mutation overlap with primary tumors. They concluded that recurrent tumors likely evolved either from residual primary disease or as a result of local selective pressures within the neural microenvironment (e.g., vascular niches, intrinsic genomic instability). Furthermore, samples arising from the primary tumor mass shared genomic and transcriptomic signatures, whereas geographically separate tumors demonstrated vastly different genomic-alteration profiles [55]. In other words, local recurrences were likely to retain similar mutational profiles with added genetic heterogeneity, while distant recurrences were more likely to have undergone divergent evolution. In terms of subtype evolution, longitudinal analysis of both the transcriptional subtype and immune microenvironment showed an increase in proneural and mesenchymal subtype composition, perhaps reflective of the chemo- and radiosensitivity of the classical subtype. The authors also saw only small changes in DNA methylation status.

Körber et al. [56] also compared genomic, epigenomic, and transcriptomic data in paired primary and recurrent glioblastoma samples following standard-of-care treatment. Their comparisons of temporally-paired samples showed mostly stable methylation status, driver mutations (e.g., *TERT* promoter, gain of chromosome 7 and/or loss of chromosome 10) and numbers of mutations between temporally paired samples. Importantly, other than DNA mismatch repair mutations—such as MSH6 and XIST—recurrent tumors shared relatively few new mutations. The replacement of mutations in oncogenic drivers (e.g., PDGFRA, EGFR, and TP53) at the time of recurrence and the development of dysregulated TGF-β signaling have also been described [57]. Most recurrent tumors appeared to derive from more than 1 clone within the original primary tumor and did not contribute additional genomic heterogeneity [53].

The Glioma Longitudinal Analysis Consortium extended this knowledge by studying a cohort of 222 patients with glioblastoma (many of whom had IDH wild type glioblastoma). The researchers found that, at recurrence, most tumors maintained the clonal structure of the original tumor and that selective pressures occurred mostly early in gliomagenesis rather than as a result of treatment [58,59]. Interestingly, treatment-induced hypermutation did not confer any positive or negative effect on patient survival. In addition, neoantigens did not undergo substantial immunoediting, suggesting that a nonsynonymous exonic mutational burden is not a significant driver of immunoediting activity.

Taken together, the findings from the above studies suggest that, although tumoral heterogeneity in glioblastomas remains mostly stable over time, subclonal mutations may result in differential drug sensitivity in a minority of cases at the time of recurrence [60]. Future efforts should continue to optimize and refine individualized treatments for patients based on their tumors’ unique mutational profiles.

### 2.3. Heterogeneity of the Tumoral Microenvironment

Intratumoral heterogeneity is generated not only by the various populations of resident cells and their intercellular communications, but also by the unique niches created by the vasculature and extracellular matrix [20]. These aggregate populations and the cross-talk molecules they share are collectively termed the tumor microenvironment (TME). Relevant cellular populations of the TME (Figure 1) include glioblastoma/glioma stem cells, tumor-infiltrating lymphocytes (TILs), tumor-associated macrophages (TAMs), myeloid-derived suppressor cells (MDSCs), and BBB cells (e.g., endothelial cells and pericytes). Intercommunication between these populations of cells occurs via secreted factors with shared signaling pathways that mediate growth, invasion, immune escape, and therapeutic resistance.

As mentioned above, recent studies support the hypothesis that the TME is partially genetically driven. Indeed, proneural (i.e., PDGFB-mutated) tumors demonstrate more permeable BBBs than mesenchymal (i.e., NF1-mutated) tumors and IDHwt gliomas harbor more monocyte-derived macrophages than microglia relative to IDHmut gliomas [51,61,62]. Transcriptomic data further suggest that proneural cells are concentrated in the leading, infiltrative edges of tumors where they use creatinine to resist the formation of reactive oxygen species via a hypoxia-inducible factor-dependent mechanism [63]. Furthermore, regression analysis-based gene deconvolution of RNA sequencing data from TCGA has also demonstrated a significant association between mesenchymal or classical composition and higher macrophage content with more negative regulation of T-cell activation [64]. Mesenchymal cells also express high levels of caspase-8, which activates NF- κB signaling in a non-canonical pathway to increase angiogenesis, growth, and transcription of factors such as VEGF and IL-8 [65,66].

In the perivascular space, a major site of cross-talk within the TME, glioblastoma cells interact with components of the BBB to promote their own survival, growth, and immune escape. Hepatocyte growth factor (HGF) secreted by tumor cells binds with c-Met to induce the transformation of endothelial cells—via Wnt/β-catenin signaling—into mesenchymal populations. These mesenchymal populations form aberrant neovasculature to promote the invasion, proliferation, and generation of GSC-maintaining hypoxic milieus [67,68]. Pericytes also maintain the TME in the perivascular spaces, in which interaction with glioblastoma cells induces an oxidative burst that promotes upregulation of the lysosome-associated membrane protein 2A (LAMP-2A) and chaperone-mediated autophagy. This leads to secretion of anti-inflammatory cytokines (e.g., IL-10, TGF-β), increased programmed death-ligand 1 (PD-L1) expression, and decreased major histocompatibility complex-II and co-stimulatory molecule expression. The net effect is the promotion of immune tolerance [66,69].

In terms of immune-cell composition, glioblastoma includes both microglia and peripherally-recruited macrophages and a smaller number of TILs. Glioblastoma cells secrete granulocyte-macrophage colony-stimulating factor (GM-CSF) to promote a shift in bone marrow hematopoiesis toward granulocytic lineages, and this causes a reduction in lymphocytic cells. Glioblastoma cells also secrete chemokines such as C-C motif chemokine 22 (CCL22) to promote regulatory T cell (T_reg_) infiltration [70,71,72]. TILs induce indoleamine 2,3 dioxygenase 1 (IDO1) expression in glioblastoma cells to promote CD25+/FoxP3+ Treg infiltration [66]. Macrophages are a major immunosuppressive cell population within the TME and have two phenotypes. The pro-inflammatory, immune-reactive phenotype is typically acquired after stimulation with toll-like receptor 4 ligands and interferon-γ, while the alternative anti-inflammatory, immune tolerant phenotype occurs after IL-4, IL-10, and IL-13 exposure and mediates immunosuppressive effects [73]. Recent work suggests that endothelial cells expressing IL-6 and microenvironmental colony-stimulating factor-1 (CSF-1) synergistically activate Akt/mTOR and contribute to a more immunosuppressive polarization [74].

Extracellular components also play an important role in maintaining the TME. To this end, glioblastoma cells secrete multiple types of molecules to enhance invasion via cell-matrix interactions, neovascularization, and growth [21,75]. One of these is tenascin-C, a glycoprotein that enhances the invasiveness of glioblastoma cells via non-adhesion and the focal-adhesion kinase pathway [76]. Fibulin-3 is another soluble glycoprotein secreted by glioblastomas that exerts effects on endothelial cells, astrocytes, and GSCs that promote growth, invasion, chemoresistance, and survival via both Notch- and NF-κB-dependent mechanisms [77,78,79]. In addition, exosomes (extracellular vesicles extruded by glioblastoma cells) contain fusion proteins that promote the mesenchymal transformation, stemness, and invasiveness of glioblastoma cells and endothelial neovascularization [80]. Recent work has identified another stromal population of human mesenchymal stem cells that secretes IL-6 and exosomal miR-1587 to promote GSC proliferation and stemness [81].

As discussed above, therapy-resistant cellular populations within the TME that promote angiogenesis, immunosuppression, and the maintenance of stemness contribute substantially to treatment resistance, and continued efforts to target these populations are warranted. The inhibition of one such axis, that of macrophage- and MDSC-related CSF-1 and its receptor, has shown promise in preclinical models of glioma, and further study is warranted to determine its therapeutic role [82,83].

## 3. Molecular Mechanisms of Resistance to Conventional Therapy

### 3.1. Resistance to Cytotoxic Chemotherapy

Although the standard post-operative treatment for newly diagnosed glioblastoma—radiation administered concurrently with the alkylating chemotherapy agent temozolomide, followed by adjuvant temozolomide—has been established for over a decade [1], glioblastoma invariably recurs and is resistant to further chemotherapy. Ionizing radiation mediates its cytotoxic effects via the induction of double-stranded and single-stranded DNA breaks, oxidative damage, mitotic cell death, and centrosome overduplication, while temozolomide induces cytotoxicity mainly by the formation of O6-methylguanine (O6MeG) adducts that cause replication-associated double-stranded DNA breaks, G2/M cell cycle arrest, and apoptosis [84,85,86].

**Figure 2:**
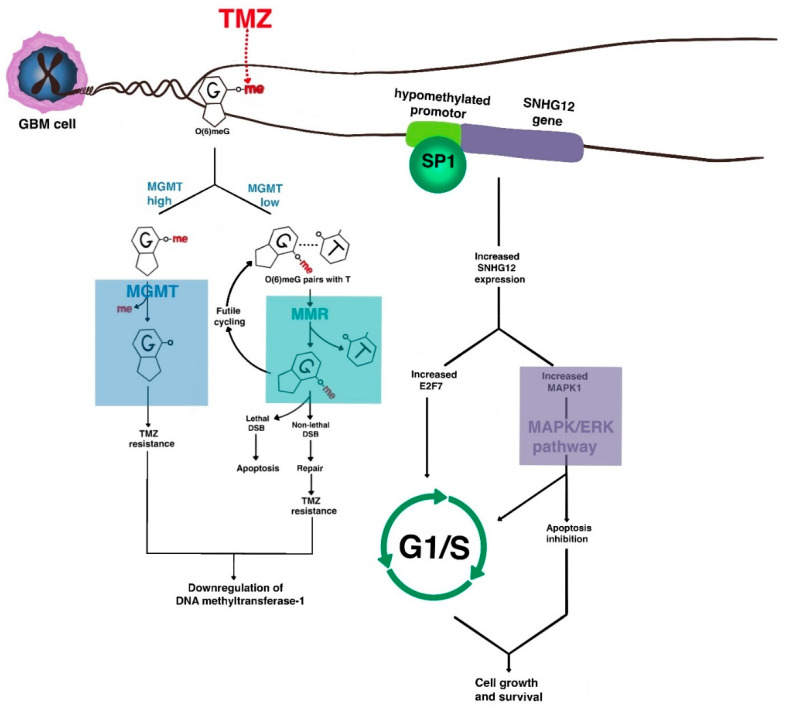
The molecular basis of chemoresistance in GBM. The major mechanisms of resistance of GBM to alkylating chemotherapy such as temozolomide (TMZ) revolve around DNA repair, cell cycle progression, and anti-apoptosis. One major resistance pathway involves the enzyme O6-MeG DNA methyltransferase (MGMT), which removes TMZ-induced O6 methyl adducts to allow DNA replication to continue. Acquired DNA mismatch repair deficiency also contributes to alkylating agent resistance. As cells acquire TMZ resistance, downregulation of DNA methyltransferase-1 occurs, leading to epigenetic de-repression of oncogenes such as SNHG12 that activate MAPK signaling to lead to inhibition of apoptosis and G1/S transition.

One of the earliest-characterized resistance mechanisms to temozolomide therapy was upregulation of the DNA repair enzyme O6MeG DNA methyltransferase (MGMT) [87], which removes the methyl adduct from DNA to allow mismatch repair and, therefore, tumoral DNA replication (Figure 2). Indeed, epigenetic silencing of MGMT via promoter hypermethylation is one of the most clinically powerful prognostic and predictive biomarkers in patients with glioblastoma [88]. Extensive work has also characterized alterations in the DNA mismatch repair system (e.g., MSH6 deficiency, MLH1 and PMS2 downregulation) that also confer resistance to temozolomide [89,90,91]. Interestingly, recent evidence has pointed to a more significant role for acquired DNA mismatch repair deficiency than for MGMT upregulation [92]. This may be because histone deacetylase 6 (HDAC6) downregulates MSH6 expression [93]. Of note, in GSCs, the predominant mechanism of DNA mismatch repair appears to be related to DNA damage response (DDR) enzymes. Therefore, targeted inhibitors of these enzymes warrant investigation as agents for chemosensitization [94,95,96].

Another recently discovered MGMT-independent mechanism involves the long non-coding RNA small nucleolar RNA host gene 12 (SNHG12), which is highly expressed in malignant gliomas via epigenetic demethylation of its promoter. The downregulation of DNA methyltransferase-1 that occurs after temozolomide resistance leads to binding of transcription factor SP1 and transcriptional activation. Cytoplasmic SNHG12 then acts as a sponge for the microRNA miR-129-5p, allowing for de-repression of MAPK1 and E2F7. This leads to anti-apoptosis and G1/S transition via the MAPK/ERK pathway [97,98]. MAPK8 is also upregulated in temozolomide-resistant cell lines [99].

NF-κB signaling has also recently been implicated in chemoresistance. EGFRvIII-expressing, temozolomide-resistant glioblastoma cells upregulate E2F6, an Rb-independent transcriptional repressor that promotes double-stranded DNA break repair [100], and tumor necrosis factor (TNF)-α secreted by TAMs regulates the conversion of proneural subtype GSCs into chemo- and radioresistant mesenchymal subtypes [101].

### 3.2. Resistance to Radiotherapy

**Figure 3:**
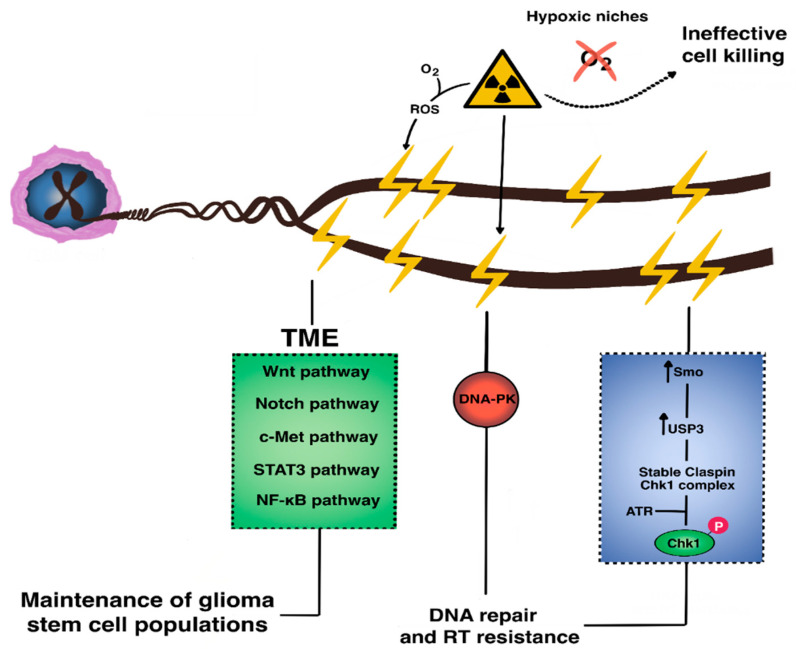
The molecular basis of radioresistance in GBM. Resistance to radiotherapy in GBM occurs via microenvironmental crosstalk across multiple signaling pathways (e.g., Wnt, Notch, c-Met, STAT3, Sonic hedgehog, and NF-κB) that collectively maintain intrinsically-radioresistant glioma stem cell populations. Within these GSCs, aberrantly upregulated DNA damage response occurs via activation of repair enzymes such as the DNA-dependent protein kinase (DNA-PK) which promotes non-homologous end joining.

The resistance of glioblastoma to radiotherapy is similarly complex (Figure 3) because of hypoxic niches that limit formation of the reactive oxygen species necessary for cell killing, hyperactivated DNA damage response machinery, and active cross-talk between TME populations via shared pathways (e.g., Wnt, Shh, Notch, c-Met, STAT3) [102,103,104]. Wnt-induced signaling protein 1 (WISP1) is significantly enriched in radioresistant glioblastoma cells and is critical in maintaining GSC survival via autocrine signaling and immunosuppressive macrophage polarization via paracrine signaling [74]. Similarly, HGF/c-Met signaling induces the expression of stem cell programming transcription factors such as SRY-box2 (SOX2), octamer-binding transcription factor 4 (OCT4), and homeobox protein Nanog (NANOG), even in differentiated cells [105,106]. The lysine methyltransferase enhancer of zeste homolog 2 (EZH2) is significantly upregulated by radiation treatment, stabilized by NIMA-related kinase 2 (NEK2) phosphorylation, and activated by mitotic kinase maternal embryonic leucine-zipper kinase (MELK), where it subsequently methylates NF-κB to maintain GSC transcriptional programs [107,108,109]. Recent work by Jeon et al. [110] demonstrated that radiation-induced senescent glioblastoma cells promote infiltration of Ly6G+ inflammatory, myeloid-derived cells that subsequently induce dedifferentiation of glioblastoma cells into resistant GSCs.

Altered DNA damage response regulation is a hallmark of GSC radioresistance. Recent work has shown that Smo, a component of the hedgehog pathway with anti-apoptotic, pro-proliferative, and pro-DNA repair functions, is overexpressed in radioresistant cells, in which it upregulates transcription of the deubiquitination enzyme ubiquitin-specific protease 3 (USP3). This enzyme stabilizes claspin, which binds Chk1 to allow it to be phosphorylated by ATR [111,112,113]. Smo knockdown in previously-irradiated recurrent glioblastoma cells leads to increased G2/M arrest and apoptosis. Replication protein A is another recently-characterized, single-stranded DNA-binding protein highly expressed in high-grade gliomas and GSCs, in which it prevents apoptosis and maintains proliferation via an ATR-dependent pathway [114,115,116].

PDZ binding kinase is a recently identified, novel serine-threonine kinase related to MAPK. It is involved in several oncogenic signaling pathways (e.g., p38, ERK1/2, focal-adhesion kinase/Src-MMP). It is highly enriched in malignant gliomas and acts as a transcriptional activator of CCNB2 expression in a radiation dose-dependent fashion to mediate resistance [117,118].

In summary, glioblastoma resistance against radio- and chemotherapy is complex and mediated across multiple signaling pathways, including the Wnt, Shh, NF-κB, DDR, and MAPK pathways. The continued study of therapies targeting these dysregulated pathways delivered to resistant GSC populations is therefore warranted.

## 4. Molecular Mechanisms of Resistance to Targeted Therapy

With the recent success of targeted therapy in a number of solid cancers and the growing knowledge of molecular alterations in glioblastoma, there have been many efforts to develop targeted therapies for the treatment of glioblastoma. Unfortunately, though isolated successes have been reported, these efforts have largely not extended into successful clinical trials. The reasons for the lack of success of targeted therapy in glioblastoma are multifold and related to intrinsic disease-related factors (e.g., inter- and intratumoral heterogeneity, signaling-pathway redundancy) and challenges related to clinical trial design (e.g., biomarker selection, difficulties with resampling intracranial tumors, brain penetration of investigational drugs).

As mentioned above, the comprehensive genomic characterization of human glioblastoma originally identified 4 glioblastoma subtypes: proneural, classical, mesenchymal, and neural, each characterized by uniquely dysregulated signaling pathways [36]. Amongst these, IDH, PDGFR, and PIK3CA alterations characterize proneural glioblastoma, EGFR amplification occurs in classical glioblastoma, and NF1, PTEN, and NFκB mutations predominate in mesenchymal subtypes. All represent attractive targets for drug development [37]. However, with the exception of IDH, these recurrent mutations have not been clearly identified as strong prognostic or predictive markers.

IDH1/2 mutations are found in about 5% of glioblastomas and are associated with longer patient survival [119,120]. Activating IDH1/2 mutations result in the overproduction of D-2-hydroxyglutarate. This in turn leads to alterations in cellular metabolism, blockade of cancer cell differentiation, and genome-wide hypermethylation and heterochromatin formation to drive tumorigenesis [121]. The activation of IDH mutations is the primary initiating event in glioma [122], but the relevance of IDH blockade in high-grade gliomas is less clear. In a recent phase I dose escalation trial of the IDH1 inhibitor ivosidenib, which included all glioma grades and enhancing as well as nonenhancing tumors, ivosidenib reduced the volume and growth rates of only nonenhancing tumors [123]. The IDH1 inhibitor vorasidenib is currently being tested in a phase III study of recurrent, low-grade glioma (NCT04164901).

Glioblastoma researchers have also targeted growth factor receptors such as EGFR and PDGFR using small-molecule tyrosine kinase inhibitors, monoclonal antibodies, and antibody drug conjugates [124], but these efforts have not yet translated into successful clinical trials. EGFR alterations (amplifications, point mutations, and rearrangements) are found in about half of glioblastomas [40]. EGFRvIII, a constitutively active variant, is seen in about 30% of all glioblastomas and has been the target of a number of investigational treatments. Despite promising preclinical and early phase clinical trials, an EGFRvIII peptide vaccine, rindopepimut, led to a negative phase III study in patients with newly diagnosed glioblastoma [125]. One main factor in the failure of this approach was the loss of EGFRvIII expression in about 60% of patients with available tumor tissue at recurrence. This loss of expression was later demonstrated in other trials targeting EGFRvIII, such as a chimeric antigen receptor (CAR) T-cell trial [126]. Other studies involving small-molecule tyrosine kinase inhibitors (e.g., geftinib, erlotinib), antibody drug conjugates (ABT-414), and monoclonal antibodies (cetuximab, nimotuzumab) similarly failed to elicit responsiveness [127,128,129,130]. The failure of these approaches is likely due to the lack of stable EGFRvIII expression during tumor evolution and the presence of redundant signaling pathways leading to resistance [131]. Isolated reports of success with targeting tyrosine kinase inhibitors less frequently altered in glioblastoma (PDGFRA, FGFR, and c-MET) have also been reported, but clinical results have been inconsistent, likely due to mechanisms of resistance similar to those seen in the EGFRvIII trials [132,133,134].

The PI3K/AKT/mTOR pathway is activated in about 30% of glioblastomas [37]. Multiple drugs targeting this pathway have been developed, but few sufficiently cross the BBB. Multiple such PI3K/AKT/mTOR-targeting agents, given as monotherapies or in combination with conventional treatments, have also failed in clinical trials [135,136]. The PI3K pathway inhibitors GDC-0068 and GDC-0084 are uniquely brain-penetrant and are currently the subject of clinical investigations (NCT02430363 and NCT03522298). Whether failure of earlier-generation PI3K inhibitors was due to poor BBB penetration or redundant signaling pathways and temporal tumor heterogeneity has not yet been determined.

Targeting the MAPK pathway has also been of interest to researchers, as a small proportion of glioblastoma and glioma subtypes harbor *BRAF* V600E mutations [137]. The RAF multikinase inhibitor sorafenib was widely studied but had limited efficacy in glioblastoma [138]. However, responses in patients with other glioma subtypes have been observed with the use of second- and third-generation BRAF inhibitors, alone or in combination with MEK inhibitors [137]. Unfortunately, only a small fraction of all glioblastomas have BRAF mutations (~3%) and could potentially benefit from these approaches.

Aberrant cell cycle progression is frequently observed in glioblastomas with TP53 and RB pathway mutations. *TP53* mutations lead to inactivating p53 mutations that cause a loss of tumor-suppressor function and glioma cell proliferation and clonal expansion. Because targeting inactivating TP53 mutations is challenging, recent efforts have focused on the inhibition of pathways that result in p53 inactivation. MDM2 and MDM4 amplifications result in p53 inactivation and MDM2 inhibition has recently emerged as a strategy to restore p53 function [139,140]. The most common RB pathway alterations are inactivation of CDKN2A/CDKN2B and RB1 and amplification of CDK4 and CDK6. Newer-generation CDK4 and CDK6 inhibitors have shown promising brain penetration and efficacy signals in brain metastases, and the results of glioblastoma trials are eagerly awaited [141,142,143]. CKD3 and CDK6 inhibitors have shown efficacy in glioblastoma models [144] and are being studied in clinical trials in glioblastoma patients (NCT02345824).

Angiogenesis, one of the hallmarks of glioblastoma pathogenesis, has been the target of therapeutic interventions. Numerous anti-angiogenic targeted therapies have been tested in glioblastoma clinical trials and have failed to improve patient survival [124]. The most well-studied anti-angiogenic therapy, bevacizumab, failed to demonstrate a survival benefit in patients with newly diagnosed glioblastoma, though as it delays disease progression and reduces the need for corticosteroids it has been approved by the FDA as a second-line therapy [4,145].

Although targeted therapy has been successful in the treatment of cancers such as EGFR- or anaplastic lymphoma kinase (ALK)-mutated non-small-cell lung cancer or BRAFV600E-mutated melanoma, targeting these mutations has been largely ineffective in glioblastoma. As we have demonstrated, many disease- and trial-specific factors contribute to this failure. Future efforts should focus on the development of preclinical models that capture the tumor heterogeneity seen in glioblastoma (glioblastoma organoids and patient-derived explants) for the robust preclinical testing of targeted therapeutics and evaluation of resistance mechanisms prior to clinical studies. In addition, close attention to the brain penetration of compounds and to well-designed, window-of-opportunity trials that allow resampling of brain tumors to evaluate for adequate drug concentration and target engagement within the tumor are needed. It is becoming apparent that single-agent targeted therapy is unlikely to produce meaningful clinical benefits in glioblastoma. Employing combinatorial approaches with or without conventional treatments (chemotherapy or radiation) may increase our chances of using our knowledge of molecular alterations in glioblastoma to develop successful therapeutic interventions.

## 5. Molecular Mechanisms of Resistance to Immunotherapy

The view that the central nervous system is an immune-privileged site in the setting of disease has grown out of favor, and many preclinical studies have established a rationale for immune-based therapies for glioblastoma. With the success of immunotherapy in the treatment of other solid malignancies and the aforementioned increased knowledge of the immunosuppressive microenvironment in glioblastoma, there has been growing interest in the development of immune-based therapies for glioblastoma [146,147]. Current immune-based therapies under investigation for glioblastoma are legion and include oncolytic virotherapy, peptide vaccination, dendritic cell vaccination, chimeric antigen receptor (CAR) T-cell therapy, and immune-checkpoint inhibition [30,125,148]. However, the results of these investigations have been largely negative. Studies focusing on immune-checkpoint inhibition given as monotherapy or in combination with the standard of care have been similarly unsuccessful [149]. The challenges of finding a suitable immune-based therapy for glioblastoma are multifaceted and largely stem from the disease’s innately immunosuppressive microenvironment (i.e., TILs, TAMs, and MDSCs).

An important aspect contributing to the immunologic “cold”-ness of glioblastoma is its capability to induce intrinsically immunosuppressive changes in patients’ immune systems. Despite its intracranial location, glioblastoma has been known to not only induce peripheral lymphopenia via bone marrow sequestration—a phenomenon associated with the loss of sphingosine-1-phosphate receptor-1 (on CD8+ T cells)—but also to interfere with proper T-cell function. Indeed, immune cells isolated from peripheral blood of patients with glioblastoma exhibit more CD4+/CD25+/FOXP3+ T_reg_ cells relative to those of patients without glioblastoma [149,150,151,152,153]. Glioblastoma cells may also induce apoptosis in lymphocytes via a FasL-dependent mechanism [154]. CD8+ TILs, moreover, express upregulated immune-checkpoint co-inhibitory molecules such as programmed death-1 (PD-1), cytotoxic T-lymphocyte-associated protein 4 (CTLA-4), T-cell immunoglobulin and mucin domain-3 (TIM-3), and lymphocyte activating 3 (LAG-3) (Figure 1), and the transcriptional profiling of these populations is reminiscent of that of hyporesponsive T cells classically exhausted by viral infections [155,156]. The net effect is an enhancement of immunosuppressive T_reg_ function. A significant portion of this may be related to genetic or epigenetic mutations [157] that lead to loss of the tumor-suppressor PTEN. In turn, this leads to the inhibition of T-cell-mediated cytotoxicity and trafficking, increased immunosuppressive cytokine expression, and autophagy inhibition via the PI3K/Akt/mTOR-dependent pathway [158,159]. Recently, single-sample gene set enrichment analysis showed that, in malignant gliomas with the poorest prognosis, *LGALS1*—which encodes galectin-1, a β-galactoside-binding protein with immunosuppressive characteristics—is highly upregulated in association with PTEN and EGFR mutations. Knockdown of *LGALS1* in glioblastoma xenografts, further, led to decreased invasiveness, proliferation, MDSCs, and immunosuppressive macrophages and cytokines (e.g., CCL2 and TGF-β) [160]. Glycoprotein A repetitions predominant (GARP) is also expressed on the surface of glioblastoma cells, where it downregulates interferon-γ production by activating CD4+ T cells [161,162].

The immunosuppressive microenvironment is maintained via the interactions between tumor cells, GSCs, TAMs, and MDSCs. GSCs secrete cytokines—such as macrophage inhibitory cytokine-1, TGF-β, and soluble CSF—which promote a switch in macrophages from a pro-inflammatory to anti-inflammatory phenotype [163]. STAT3 signaling in TAMs is critical to this maintenance of immune tolerance to neoplastic antigens and to the favoring of Th17-predominant responses with TILs [164,165]. Targeted inhibition of STAT3 signaling reverses immune tolerance and promotes cytotoxicity even in temozolomide-resistant glioblastoma [166,167,168]. GSCs also express less toll-like receptor 4 (TLR4), which normally signals to activate transcriptional factor retinoblastoma binding protein 5 (RBBP5) to decrease the expression of stem-cell-maintenance genes, including *SOX2*, *OCT4*, and *NANOG* [169]. The role of MDSCs is increasingly appreciated. Comprising a heterogeneous group of myeloid-derived precursor cells at different stages of differentiation that express CD11b and CD33 but no mature lymphoid or myeloid markers, MDSCs are highly enriched in progressive glioblastoma, likely via the secretion of a number of factors (e.g., IL-6, IL-10, VEGF, and TGF-β) [170,171,172,173,174]. MDSCs also secrete reactive oxygen and nitrogen species to induce apoptosis of natural killer and activated T cells, express IDO to deplete tryptophan and consequently impair cytotoxic T-cell responses, promote expansion of immunosuppressive T_reg_ populations, and—importantly—promote immunosuppressive macrophage polarization by cell–cell contact [175,176,177]. A recent study has further elucidated the role of a non-receptor tyrosine kinase, Fyn, downstream of several crucial signaling pathways, including c-MET, EGFR, STAT3, and PIK3/Akt, not only in promoting the trafficking and expansion of MDSCs within the glioblastoma microenvironment, but also in promoting CD8+ T-cell exhaustion [178].

Despite their successes in other solid malignancies, immune-checkpoint inhibitors such as those targeting the PD-1/PD-L1 axis have largely failed in large-scale clinical trials for glioblastoma. This is most likely attributable to the aforementioned immunosuppressive microenvironment and the low expression of PD-1 and CTLA-4 ligands (e.g., CD274, PDCD1LG2, CD80, CD86) [16]. Indeed, among the known biomarkers that predict benefit from checkpoint inhibitors, the mutational burden is high in less than 4%, DNA mismatch repair defects (e.g., mutations in MSH2, MLH1, MSH6, and PMS2) are infrequent, and microsatellite instability is almost never detected [59,179,180,181]. Despite the high numbers of CD8+ T cells seen in tumors that acquired hypermutation in response to temozolomide, patients with temozolomide-induced hypermutation do not seem to benefit from checkpoint inhibition, which supports the hypothesis that the clonal neoantigens generated by earlier events in gliomagenesis are mostly responsible for inducing T-cell immune responses [182]. Interestingly, MAPK pathway alterations such as PTPN11 and BRAF mutations are enriched in patients who respond to anti-PD-1 therapy, although these represent only a small proportion of patients with glioblastoma [183]. Patients with glioblastoma in a recent phase II trial of pre-operative nivolumab were found to have upregulation of immune-related transcripts, including CXCL10, CCL4, and CCL31L1, and downregulation of CRP, SSx4, and CR2. However, expression of the T-cell activation marker CD137 was rarely seen [184]. Taken together, these findings show that most patients with glioblastoma are unlikely to benefit from checkpoint-inhibitor monotherapy.

Of note, the standard chemotherapeutic treatment of malignant glioma induces immunosuppression that may interfere with immune-based therapies. Dexamethasone is a critical medication for managing the symptoms of vasogenic edema, but it also depresses T-cell proliferation and induces CTLA-4 expression in CD4+ and CD8+ cells [185]. In a mouse model, administration of systemic bis-chloroethylnitrosourea induced lymphodepletion, decreased the number of TILs, and reduced the survival benefit from PD-1 blockade [186]. Furthermore, during concurrent chemoradiation with temozolomide, the absolute T and B cell counts were reduced and remained low even after the completion of treatment, and the composition of these immune cells shifted toward persistent increased Treg expression with concomitant decreased expression of naïve CD4+ T cells [187]. Interestingly, PD-1/PD-L1 checkpoint inhibition in temozolomide-resistant tumors also led to increased immunosuppressive macrophage infiltration in tumors [188] which was resolved by the co-administration of a PI3Kγ inhibitor.

Cell-mediated immunotherapy strategies such CAR T-cell therapy also carry unique challenges. Despite significant initial enthusiasm after a reported success in a single patient with multifocal glioblastoma who received CAR T-cell therapy directed against IL13Rα2 [189], subsequent studies have not borne similar results, although it should be noted that these were administered to the target organ directly via the cerebrospinal fluid rather than peripherally infused as was the case in subsequent trials. In a study of CAR T cells directed against EGFRvIII, a similarly high degree of CD4+/CD25+/FoxP3+ T cells were found to have infiltrated the tumor after CAR T-cell infusion, with upregulation of immunosuppressive molecules (e.g., IDO1, PD-L1, IL-10) [126]. Encouragingly, vaccination strategies have induced significant infiltration of differentiated and cytotoxic CD8+ T cells with only a small minority of CD4+ Treg cells [190], although a statistically significant survival benefit remains to be proven [191,192]. Finally, oncolytic virus strategies have also induced immunogenic tumor cell death and alteration of the tumor immune microenvironment with a shift toward immunosuppressive macrophage polarization and sustained type I interferon-dominant responses [193,194,195]. Preclinical evidence further supports the potential efficacy of combined oncolytic virus strategies and immune-checkpoint inhibition [196].

From the above discussion, it is reasonable to posit that any immune-based therapy, whether combined with conventional or novel therapies or given as monotherapy should ideally target the immune-cell compositional changes that are likely to thwart efficacy. For example, Wu et al. [197] found that targeting chemokine receptor CXCR4—which is overexpressed in glioblastoma and associated with a poor prognosis—in addition to PD-1 led to a significant decrease in MDSCs and increased circulating inflammatory anti-tumoral cytokines such as interferon-γ and TNF-α. Future studies should focus on such rationally combined treatments to maximize the chances of success.

## 6. Conclusions

Although glioblastoma remains one of the deadliest primary central nervous system malignancies and is treated in a manner that has not been improved upon in nearly 2 decades, lessons from various treatment failures continue to spur efforts to improve survival in patients with these tumors. Going forward, it is clear that practice-changing therapies will need to be individualized to take into account the unique genomic, epigenomic, immunologic, and microenvironmental characteristics of each tumor.

## Data Availability

No new data were created or analyzed in this study. Data sharing is not applicable to this article.

## References

[1] Stupp R., Mason W.P., van den Bent M.J., Weller M., Fisher B., Taphoorn M.J.B., Belanger K., Brandes A.A., Marosi C., Bogdahn U. (2005). Radiotherapy plus Concomitant and Adjuvant Temozolomide for Glioblastoma. N. Engl. J. Med..

[2] Westphal M., Hilt D.C., Bortey E., Delavault P., Olivares R., Warnke P.C., Whittle I.R., Jääskeläinen J., Ram Z. (2003). A phase 3 trial of local chemotherapy with biodegradable carmustine (BCNU) wafers (Gliadel wafers) in patients with primary malignant glioma. Neuro Oncol..

[3] Kreisl T.N., Kim L., Moore K., Duic P., Royce C., Stroud I., Garren N., Mackey M., Butman J.A., Camphausen K. (2009). Phase II Trial of Single-Agent Bevacizumab Followed by Bevacizumab Plus Irinotecan at Tumor Progression in Recurrent Glioblastoma. J. Clin. Oncol..

[4] Chinot O.L., Wick W., Mason W., Henriksson R., Saran F., Nishikawa R., Carpentier A.F., Hoang-Xuan K., Kavan P., Cernea D. (2014). Bevacizumab plus Radiotherapy–Temozolomide for Newly Diagnosed Glioblastoma. New Engl. J. Med..

[5] Stupp R., Taillibert S., Kanner A.A., Kesari S., Steinberg D.M., Toms S.A., Taylor L.P., Lieberman F., Silvani A., Fink K.L. (2015). Maintenance Therapy with Tumor-Treating Fields Plus Temozolomide vs Temozolomide Alone for Glioblastomaa: A Randomized Clinical Trial. JAMA.

[6] Ballabh P., Braun A., Nedergaard M. (2004). The blood–brain barrier: An overview: Structure, regulation, and clinical implica-tions. Neurobiol. Dis..

[7] Daneman R., Prat A. (2015). The Blood–Brain Barrier. Cold Spring Harb. Perspect. Biol..

[8] Pappenheimer J.R., Renkin E.M., Borrero L.M. (1951). Filtration, Diffusion and Molecular Sieving Through Peripheral Capillary Membranes; a con-tribution to the pore theory of capillary permeability. Am. J. Physiol. Content.

[9] Ueda K., Cornwell M.M., Gottesman M.M., Pastan I., Roninson I.B., Ling V., Riordan J.R. (1986). The mdrl gene, responsible for multidrug-resistance, codes for P-glycoprotein. Biochem. Biophys. Res. Commun..

[10] Cole S.P., Bhardwaj G., Gerlach J.H., E Mackie J., E Grant C., Almquist K.C., Stewart A.J., Kurz E.U., Duncan A.M., Deeley R.G. (1992). Overexpression of a transporter gene in a multidrug-resistant human lung cancer cell line [published correction appears in Science]. Science.

[11] Tsuji A., Terasaki T., Takabatake Y., Tenda Y., Tamai I., Yamashima T., Moritani S., Tsuruo T., Yamashita J. (1992). P-glycoprotein as the drug efflux pump in primary cultured bovine brain capillary endothelial cells. Life Sci..

[12] Becker C.M., Oberoi R.K., McFarren S.J., Muldoon D.M., Pafundi D.H., Pokorny J.L., Brinkmann D.H., Ohlfest J.R., Sarkaria J.N., Largaespada D.A. (2015). Decreased affinity for efflux transporters increases brain penetrance and molecular targeting of a PI3K/mTOR inhibitor in a mouse model of glioblastoma. Neuro Oncol..

[13] Kizilbash S.H., Gupta S.K., Chang K., Kawashima R., Parrish K.E., Carlson B.L., Bakken K.K., Mladek A.C., Schroeder M.A., Decker P.A. (2017). Restricted Delivery of Talazoparib Across the Blood–Brain Barrier Limits the Sensitizing Effects of PARP Inhibition on Temozolomide Therapy in Glioblastoma. Mol. Cancer.

[14] Cheng L., Huang Z., Zhou W., Wu Q., Donnola S., Liu J.K., Fang X., Sloan A.E., Mao Y., Lathia J.D. (2013). Glioblastoma Stem Cells Generate Vascular Pericytes to Support Vessel Function and Tumor Growth. Cell.

[15] Ratnam N.M., Gilbert M.R., Giles A.J. (2018). Immunotherapy in CNS cancers: The role of immune cell trafficking. Neuro Oncol..

[16] Darmanis S., Sloan S.A., Croote D., Mignardi M., Chernikova S., Samghababi P., Zhang Y., Neff N., Kowarsky M., Caneda C. (2017). Single-Cell RNA-Seq Analysis of Infiltrating Neoplastic Cells at the Migrating Front of Human Glioblastoma. Cell Rep..

[17] Zhao C., Wang H., Xiong C., Liu Y. (2018). Hypoxic glioblastoma release exosomal VEGF-A induce the permeability of blood-brain barrier. Biochem. Biophys. Res. Commun..

[18] Wang W., Dentler W.L., Borchardt R.T. (2001). VEGF increases BMEC monolayer permeability by affecting occludin expression and tight junction assembly. Am. J. Physiol. Circ. Physiol..

[19] Singh S.K., Hawkins C., Clarke I.D., Squire J.A., Bayani J., Hide T., Henkelman R.M., Cusimano M.D., Dirks P.B. (2004). Identification of human brain tumour initiating cells. Nat. Cell Biol..

[20] Calabrese C., Poppleton H., Kocak M., Hogg T.L., Fuller C., Hamner B., Oh E.Y., Gaber M.W., Finklestein D., Allen M. (2007). A Perivascular Niche for Brain Tumor Stem Cells. Cancer Cell.

[21] Mettang M., Meyer-Pannwitt V., Karpel-Massler G., Zhou S., Carragher N.O., Föhr K.J., Baumann B., Nonnenmacher L., Enzenmüller S., Dahlhaus M. (2018). Blocking distinct interactions between Glioblastoma cells and their tissue microenvironment: A novel multi-targeted therapeutic approach. Sci. Rep..

[22] Guryanova O.A., Wu Q., Cheng L., Lathia J.D., Huang Z., Yang J., MacSwords J., Eyler C.E., McLendon R.E., Heddleston J.M. (2011). Nonreceptor Tyrosine Kinase BMX Maintains Self-Renewal and Tumorigenic Potential of Glioblastoma Stem Cells by Activating STAT3. Cancer Cell.

[23] Zhou W., Chen C., Shideng B., Bian X., Gimple R.C., Fang X., Huang Z., Zhai K., Ke S.Q., Ping Y.-F. (2017). Targeting Glioma Stem Cell-Derived Pericytes Disrupts the Blood-Tumor Barrier and Improves Chemotherapeutic Efficacy. Cell Stem Cell.

[24] Shi Y., Guryanova O.A., Zhou W., Liu C., Huang Z., Fang X., Wang X., Chen C., Wu Q., He Z. (2018). Ibrutinib inactivates BMX-STAT3 in glioma stem cells to impair malignant growth and radioresistance. Sci. Transl. Med..

[25] Alvarez J.I., Dodelet-Devillers A., Kebir H., Ifergan I., Fabre P.J., Terouz S., Sabbagh M., Wosik K., Bourbonnière L., Bernard M. (2011). The Hedgehog Pathway Promotes Blood-Brain Barrier Integrity and CNS Immune Quiescence. Science.

[26] Findley M.K., Koval M. (2009). Regulation and roles for claudin-family tight junction proteins. IUBMB Life.

[27] Roudnicky F., Kim B.K., Lan Y., Schmucki R., Küppers V., Christensen K., Graf M., Patsch C., Burcin M., Meyer C.A. (2020). Identification of a combination of transcription factors that synergistically increases endothelial cell barrier resistance. Sci. Rep..

[28] Chang J., Mancuso M.R., Maier C., Liang X., Yuki K., Yang L., Kwong J.W., Wang J., Rao V., Vallon M. (2017). Gpr124 is essential for blood–brain barrier integrity in central nervous system disease. Nat. Med..

[29] Griveau A., Seano G., Shelton S.J., Kupp R., Jahangiri A., Obernier K., Krishnan S., Lindberg O.R., Yuen T.J., Tien A.-C. (2018). A Glial Signature and Wnt7 Signaling Regulate Glioma-Vascular Interactions and Tumor Microenvironment. Cancer Cell.

[30] Desjardins A., Gromeier M., Ii J.E.H., Beaubier N., Bolognesi D.P., Friedman A.H., Friedman H.S., McSherry F., Muscat A., Nair S. (2018). Recurrent Glioblastoma Treated with Recombinant Poliovirus. New Engl. J. Med..

[31] Bogdahn U., Hau P., Stockhammer G., Venkataramana N.K., Mahapatra A.K., Suri A., Balasubramaniam A., Nair S., Oliushine V., Parfenov V. (2010). Targeted therapy for high-grade glioma with the TGF- 2 inhibitor trabedersen: Results of a randomized and controlled phase IIb study. Neuro Oncol..

[32] Kunwar S., Chang S.M., Westphal M., Vogelbaum M., Sampson J., Barnett G., Shaffrey M., Ram Z., Piepmeier J., Prados M. (2010). Phase III randomized trial of CED of IL13-PE38QQR vs Gliadel wafers for recurrent glioblastoma. Neuro Oncol..

[33] Chen K.-T., Lin Y.-J., Chai W.-Y., Lin C.-J., Chen P.-Y., Huang C.-Y., Kuo J.S., Liu H.-L., Wei K.-C. (2020). Neuronavigation-guided focused ultrasound (NaviFUS) for transcranial blood-brain barrier opening in recurrent glioblastoma patients: Clinical trial protocol. Ann. Transl. Med..

[34] Thomas E., Colombeau L., Gries M., Peterlini T., Mathieu C., Thomas N., Boura C., Frochot C., Vanderesse R., Lux F. (2017). Ultrasmall AGuIX theranostic nanoparticles for vascular-targeted interstitial photodynamic therapy of glioblastoma. Int. J. Nanomed..

[35] Pandey V., Haider T., Chandak A.R., Chakraborty A., Banerjee S., Soni V. (2020). Surface modified silk fibroin nanoparticles for improved delivery of doxorubicin: Development, characterization, in-vitro studies [published online ahead of print, 2020 August 3]. Int. J. Biol. Macromol..

[36] (2008). The Cancer Genome Atlas Research Network Comprehensive genomic characterization defines human glioblastoma genes and core pathways [published correction appears in Nature]. Nat. Cell Biol..

[37] Verhaak R.G., Hoadley K.A., Purdom E., Wang V., Qi Y., Wilkerson M.D., Miller C.R., Ding L., Golub T., Mesirov J.P. (2010). Integrated Genomic Analysis Identifies Clinically Relevant Subtypes of Glioblastoma Characterized by Abnormalities in PDGFRA, IDH1, EGFR, and NF1. Cancer Cell.

[38] Noushmehr H., Weisenberger D.J., Diefes K., Phillips H.S., Pujara K., Berman B.P., Pan F., Pelloski C.E., Sulman E.P., Bhat K.P. (2010). Identification of a CpG Island Methylator Phenotype that Defines a Distinct Subgroup of Glioma. Cancer Cell.

[39] Kim Y.-W., Koul D., Kim S.H., Lucio-Eterovic A.K., Freire P.R., Yao J., Wang J., Almeida J.S., Aldape K., Yung W.A. (2013). Identification of prognostic gene signatures of glioblastoma: A study based on TCGA data analysis. Neuro Oncol..

[40] Brennan C.W., Verhaak R.G.W., McKenna A., Campos B., Noushmehr H., Salama S.R., Zheng S., Chakravarty D., Sanborn J.Z., Berman S.H. (2013). The Somatic Genomic Landscape of Glioblastoma. Cell.

[41] Wang Q., Hu X., Hu B., Muller F., Kim H., Squatrito M., Millelsen T., Scarpace L., Barthel F., Lin Y.-H. (2016). Tumor evolution of glioma intrinsic gene expression subtype associates with immunological changes in the microenvironment. bioRxiv.

[42] Gill B.J., Pisapia D.J., Malone H.R., Goldstein H., Lei L., Sonabend A., Yun J., Samanamud J., Sims J.S., Banu M. (2014). MRI-localized biopsies reveal subtype-specific differences in molecular and cellular composition at the margins of glioblastoma. Proc. Natl. Acad. Sci. USA.

[43] Neftel C., Laffy J., Filbin M.G., Hara T., Shore M.E., Rahme G.J., Richman A.R., Silverbush D., Shaw M.L., Hebert C.M. (2019). An Integrative Model of Cellular States, Plasticity, and Genetics for Glioblastoma. Cell.

[44] Sottoriva A., Spiteri I., Piccirillo S.G.M., Touloumis A., Collins V.P., Marioni J.C., Curtis C., Watts C., Tavaré S. (2013). Intratumor heterogeneity in human glioblastoma reflects cancer evolutionary dynamics. Proc. Natl. Acad. Sci. USA.

[45] Patel A.P., Tirosh I., Trombetta J.J., Shalek A.K., Gillespie S.M., Wakimoto H., Cahill D.P., Nahed B.V., Curry W.T., Martuza R.L. (2014). Single-cell RNA-seq highlights intratumoral heterogeneity in primary glioblastoma. Science.

[46] Rennert R., Achrol A.S., Januszyk M., Kahn S.A., Liu T.T., Liu Y., Sahoo D., Rodrigues M., Maan Z.N., Wong V.W. (2016). Multiple Subsets of Brain Tumor Initiating Cells Coexist in Glioblastoma a: Multiplici-ty Exists Within GBM Initiating Cells. Stem Cells.

[47] Pesenti C., Navone S.E., Guarnaccia L., Terrasi A., Costanza J., Silipigni R., Guarneri S., Fusco N., Fontana L., Locatelli M. (2019). The Genetic Landscape of Human Glioblastoma and Matched Primary Cancer Stem Cells Reveals Intratumour Similarity and Intertumour Heterogeneity. Stem Cells Int..

[48] Piccirillo S., Colman S., Potter N.E., Van Delft F.W., Lillis S., Carnicer M.-J., Kearney L., Watts C., Greaves M. (2014). Genetic and functional diversity of propagating cells in glioblastoma. Stem Cell Rep..

[49] Meyer M., Reimand J., Lan X., Head R., Zhu X., Kushida M., Bayani J., Pressey J.C., Lionel A.C., Clarke I.D. (2015). Single cell-derived clonal analysis of human glioblastoma links functional and genomic heterogeneity. Proc. Natl. Acad. Sci. USA.

[50] Skaga E., Kulesskiy E., Fayzullin A., Sandberg C.J., Potdar S., Kyttälä A., Langmoen I.A., Laakso A., Gaál-Paavola E., Perola M. (2019). Intertumoral heterogeneity in patient-specific drug sensitivities in treatment-naïve glioblastoma. BMC Cancer.

[51] Herting C.J., Chen Z., Pitter K.L., Szulzewsky F., Kaffes I., Kaluzova M., Park J.C., Cimino P.J., Brennan C., Wang B. (2017). Genetic driver mutations define the expression signature and microenvironmental composition of high-grade gliomas. Glia.

[52] Jacob F., Salinas R.D., Zhang D.Y., Nguyen P.T., Schnoll J.G., Wong S.Z.H., Thokala R., Sheikh S., Saxena D., Prokop S. (2020). A Patient-Derived Glioblastoma Organoid Model and Biobank Recapitulates Inter- and Intra-tumoral Heterogeneity. Cell.

[53] Kim H., Zheng S., Amini S.S., Virk S.M., Mikkelsen T., Brat D.J., Grimsby J., Sougnez C., Muller F., Hu J. (2015). Whole-genome and multisector exome sequencing of primary and post-treatment glioblastoma reveals patterns of tumor evolution. Genome Res..

[54] Schäfer N., Gielen G.H., Rauschenbach L., Kebir S., Till A., Reinartz R., Simon M., Niehusmann P., Kleinschnitz C., Herrlinger U. (2019). Longitudinal heterogeneity in glioblastoma: Moving targets in recurrent versus primary tumors. J. Transl. Med..

[55] Kim J., Lee I.-H., Cho H.J., Park C.-K., Jung Y.-S., Kim Y., Nam S.H., Kim B.S., Johnson M.D., Kong D.-S. (2015). Spatiotemporal Evolution of the Primary Glioblastoma Genome. Cancer Cell.

[56] Körber V., Yang J., Barah P., Wu Y., Stichel D., Gu Z., Fletcher M.N.C., Jones D., Hentschel B., Lamszus K. (2019). Evolutionary Trajectories of IDHWT Glioblastomas Reveal a Common Path of Early Tumorigenesis Instigated Years ahead of Initial Diagnosis. Cancer Cell.

[57] Wang J., Cazzato E., Ladewig E., Frattini V., Rosenbloom D.I.S., Zairis S., Abate F., Liu Z., Elliott O., Shin Y.-J. (2016). Clonal evolution of glioblastoma under therapy. Nat. Genet..

[58] Akgül S., Patch A.-M., D’Souza R.C.J., Mukhopadhyay P., Nones K., Kempe S., Kazakoff S., Jeffree R.L., Stringer B.W., Pearson J.V. (2019). Intratumoural Heterogeneity Underlies Distinct Therapy Responses and Treatment Resistance in Glioblastoma. Cancers.

[59] Barthel F.P., Johnson K.C., Varn F.S., Moskalik A.D., Tanner G., Kocakavuk E., Anderson K.J., Abiola O., Aldape K., The GLASS Consortium (2019). Longitudinal molecular trajectories of diffuse glioma in adults. Nature.

[60] Kim E.L., Sorokin M., Kantelhardt S.R., Kalasauskas D., Sprang B., Fauss J., Ringel F., Garazha A., Albert E., Gaifullin N.M. (2020). Intratumoral Heterogeneity and Longitudinal Changes in Gene Expression Predict Differential Drug Sensitivity in Newly Diagnosed and Recurrent Glioblastoma. Cancers.

[61] Klemm F., Maas R.R., Bowman R.L., Kornete M., Soukup K., Nassiri S., Brouland J.-P., Iacobuzio-Donahue C.A., Brennan C., Tabar V. (2020). Interrogation of the Microenvironmental Landscape in Brain Tumors Reveals Disease-Specific Alterations of Immune Cells. Cell.

[62] Friebel E., Kapolou K., Unger S., Núñez N.G., Utz S., Rushing E.J., Regli L., Weller M., Greter M., Tugues S. (2020). Single-Cell Mapping of Human Brain Cancer Reveals Tumor-Specific Instruction of Tissue-Invading Leukocytes. Cell.

[63] Heiland D.H., Gaebelein A., Boerries M., Woerner J., Pompe N., Franco P., Heynckes S., Bartholomä M.D., Hailín D.Ó., Carro M.S. (2018). Microenvironment-Derived Regulation of HIF Signaling Drives Transcriptional Heterogeneity in Glioblastoma Multiforme. Mol. Cancer Res..

[64] Luoto S., Hermelo I., Vuorinen E.M., Hannus P., Kesseli J., Nykter M., Granberg K.J. (2018). Computational Characterization of Suppressive Immune Microenvironments in Glioblastoma. Cancer Res..

[65] Fianco G., Mongiardi M.P., Levi A., De Luca T., Desideri M., Trisciuoglio D., Del Bufalo D., Cinà I., Di Benedetto A., Mottolese M. (2017). Caspase-8 contributes to angiogenesis and chemotherapy resistance in glioblastoma. eLife.

[66] Valdor R., García-Bernal D., Bueno C., Ródenas M., Moraleda J.M., Macian F., Martínez S. (2017). Glioblastoma progression is assisted by induction of immunosuppressive function of pericytes through interaction with tumor cells. Oncotarget.

[67] Huang M., Liu T., Ma P., Mitteer R.A., Zhang Z., Kim H.J., Yeo E., Zhang D., Cai P., Li C. (2016). c-Met–mediated endothelial plasticity drives aberrant vascularization and chemoresistance in glioblastoma. J. Clin. Investig..

[68] Huang M., Zhang D., Wu J.Y., Xing K., Yeo E., Li C., Zhang L., Holland E.C., Yao L., Qin L. (2020). Wnt-mediated endothelial transformation into mesenchymal stem cell–like cells induces chemoresistance in glioblastoma. Sci. Transl. Med..

[69] Valdor R., García-Bernal D., Riquelme D., Martínez C.M., Moraleda J.M., Cuervo A.M., Macian F., Martínez S. (2019). Glioblastoma ablates pericytes antitumor immune function through aberrant up-regulation of chaperone-mediated autophagy. Proc. Natl. Acad. Sci. USA.

[70] Kast R.E., A Hill Q., Wion D., Mellstedt H., Focosi D., Karpel-Massler G., Heiland T., Halatsch M.-E. (2017). Glioblastoma-synthesized G-CSF and GM-CSF contribute to growth and immunosuppression: Potential therapeutic benefit from dapsone, fenofibrate, and ribavirin. Tumor Biol..

[71] Zhai L., Ladomersky E., Lauing K.L., Wu M., Genet M., Gritsina G., Győrffy B., Brastianos P.K., Binder D.C., Sosman J.A. (2017). Infiltrating T Cells Increase IDO1 Expression in Glioblastoma and Contribute to Decreased Patient Survival. Clin. Cancer Res..

[72] Crane C.A., Ahn B.J., Han S.J., Parsa A.T. (2012). Soluble factors secreted by glioblastoma cell lines facilitate recruitment, survival, and expansion of regulatory T cells: Implications for immunotherapy. Neuro Oncol..

[73] Mantovani A., Sozzani S., Locati M., Allavena P., Sica A. (2002). Macrophage polarization: Tumor-associated macrophages as a paradigm for polarized M2 mononuclear phagocytes. Trends Immunol..

[74] Wang Q., He Z., Huang M., Liu T., Wang Y., Xu H., Duan H., Ma P., Zhang L., Zamvil S.S. (2018). Vascular niche IL-6 induces alternative macrophage activation in glioblastoma through HIF-2α. Nat. Commun..

[75] Westhoff M.-A., Zhou S., Nonnenmacher L., Karpel-Massler G., Jennewein C., Schneider M., Halatsch M.-E., Carragher N.O., Baumann B., Krause A. (2013). Inhibition of NF- B Signaling Ablates the Invasive Phenotype of Glioblastoma. Mol. Cancer Res..

[76] Xia S., Lal B., Tung B., Wang S., Goodwin C.R., Laterra J. (2016). Tumor microenvironment tenascin-C promotes glioblastoma invasion and negatively regulates tumor proliferation. Neuro Oncol..

[77] Nandhu M.S., Kwiatkowska A., Bhaskaran V., Hayes J., Hu B., Viapiano M.S. (2017). Tumor-derived fibulin-3 activates pro-invasive NF-κB signaling in glioblastoma cells and their microenvironment. Oncogene.

[78] Hu B., Nandhu M.S., Sim H., Agudelo-Garcia P.A., Saldivar J.C., Dolan C.E., Mora M.E., Nuovo G.J., Cole S.E., Viapiano M.S. (2012). Fibulin-3 Promotes Glioma Growth and Resistance through a Novel Paracrine Regulation of Notch Signaling. Cancer Res..

[79] Hiddingh L., Tannous B.A., Teng J., Tops B.B., Jeuken J., Hulleman E., Boots-Sprenger S.H., Vandertop W.P., Noske D.P., Kaspers G.J. (2013). EFEMP1 induces γ-secretase/Notch-mediated temozolomide resistance in glioblastoma. Oncotarget.

[80] Zeng A.-L., Yan W., Liu Y.-W., Wang Z., Hu Q., Nie E., Zhou X., Li R., Wang X.-F., Jiang T. (2017). Tumour exosomes from cells harbouring PTPRZ1–MET fusion contribute to a malignant phenotype and temozolomide chemoresistance in glioblastoma. Oncogene.

[81] Hossain A., Gumin J., Gao F., Figueroa J., Shinojima N., Takezaki T., Priebe W., Villarreal D., Kang S.-G., Joyce C. (2015). Mesenchymal Stem Cells Isolated from Human Gliomas Increase Proliferation and Maintain Stemness of Glioma Stem Cells Through the IL-6/gp130/STAT3 Pathway. Stem Cells.

[82] Akkari L., Bowman R.L., Tessier J., Klemm F., Handgraaf S.M., De Groot M., Quail D.F., Tillard L., Gadiot J., Huse J.T. (2020). Dynamic changes in glioma macrophage populations after radiotherapy reveal CSF-1R inhibition as a strategy to overcome resistance. Sci. Transl. Med..

[83] Pyonteck S.M., Akkari L., Schuhmacher A.J. (2013). CSF-1R inhibition alters macrophage polarization and blocks glioma progression. Nat. Med..

[84] Sato N., Mizumoto K., Nakamura M. (2000). Radiation-induced centrosome overduplication and multiple mitotic spindles in human tumor cells. Exp. Cell. Res..

[85] Fu D., Calvo J.A., Samson L.D. (2012). Balancing repair and tolerance of DNA damage caused by alkylating agents. Nat. Rev. Cancer.

[86] Roos W.P., Batista L.F.Z., Naumann S.C., Wick W., Weller M., Menck C.F.M., Kaina B. (2006). Apoptosis in malignant glioma cells triggered by the temozolomide-induced DNA lesion O6-methylguanine. Oncogene.

[87] Kitange G.J., Carlson B.L., Schroeder M.A., Grogan P.T., Lamont J.D., Decker P.A., Wu W., James C.D., Sarkaria J.N. (2009). Induction of MGMT expression is associated with temozolomide resistance in glioblastoma xenografts. Neuro Oncol..

[88] Hegi M.E., Diserens A.-C., Gorlia T. (2005). MGMT gene silencing and benefit from temozolomide in glioblastoma. N. Engl. J. Med..

[89] Shinsato Y., Furukawa T., Yunoue S. (2013). Reduction of mlh1 and pms2 confers temozolomide resistance and is associated with recurrence of glioblastoma. Oncotarget.

[90] Cahill D.P., Levine K.K., Betensky R.A. (2007). Loss of the mismatch repair protein MSH6 in human glioblastomas is associated with tumor progression during temozolomide treatment. Clin. Cancer Res..

[91] Stark A.M., Doukas A., Hugo H.-H., Hedderich J., Hattermann K., Mehdorn H.M., Held-Feindt J. (2014). Expression of DNA mismatch repair proteins MLH1, MSH2, and MSH6 in recurrent glioblastoma. Neurol. Res..

[92] Yamashiro K., Nakao K., Ohba S., Hirose Y. (2020). Human Glioma Cells Acquire Temozolomide Resistance After Repeated Drug Exposure Via DNA Mismatch Repair Dysfunction. Anticancer. Res..

[93] Kim G.W., Lee D.H., Yeon S.-K., Jeon Y.H., Yoo J., Lee S.W., Kwon S.H. (2019). Temozolomide-resistant Glioblastoma Depends on HDAC6 Activity Through Regulation of DNA Mismatch Repair. Anticancer. Res..

[94] Chen J., Li Y., Yu T.-S., McKay R.M., Burns D.K., Kernie S.G., Parada L.F. (2012). A restricted cell population propagates glioblastoma growth after chemotherapy. Nat. Cell Biol..

[95] Wang Y., Xu H., Liu T., Huang M., Butter P.-P., Li C., Zhang L., Kao G.D., Gong Y., Maity A. (2018). Temporal DNA-PK activation drives genomic instability and therapy resistance in glioma stem cells. JCI Insight.

[96] Bao S., Wu Q., McLendon R.E., Hao Y., Shi Q., Hjelmeland A.B., Dewhirst M.W., Bigner D.D., Rich J.N. (2006). Glioma stem cells promote radioresistance by preferential activation of the DNA damage response. Nat. Cell Biol..

[97] Chang K.-Y., Hsu T.-I., Hsu C.-C., Tsai S.-Y., Liu J.-J., Chou S.-W., Liu M.-S., Liou J.-P., Ko C.-Y., Chen K.-Y. (2017). Specificity protein 1-modulated superoxide dismutase 2 enhances temozolomide resistance in glioblastoma, which is independent of O 6 -methylguanine-DNA methyltransferase. Redox Biol..

[98] Lu C., Wei Y., Wang X., Zhang Z., Yin J., Li W., Chen L., Lyu X., Shi Z., Yan W. (2020). DNA-methylation-mediated activating of lncRNA SNHG12 promotes temozolomide resistance in glioblastoma. Mol. Cancer.

[99] Xu P., Zhang G., Hou S.-X., Sha L.-G. (2018). MAPK8 mediates resistance to temozolomide and apoptosis of glioblastoma cells through MAPK signaling pathway. Biomed. Pharm..

[100] Kim Y., Kim K.H., Lee J., Lee Y.-A., Kim M., Lee S.J., Park K., Yang H., Jin J., Joo K.M. (2011). Wnt activation is implicated in glioblastoma radioresistance. Lab. Investig..

[101] Huang K., Liu X., Li Y., Wang Q., Zhou J., Wang Y., Dong F., Yang C., Sun Z., Fang C. (2019). Genome-Wide CRISPR-Cas9 Screening Identifies NF-κB/E2F6 Responsible for EGFRvIII-Associated Temozolomide Resistance in Glioblastoma. Adv. Sci..

[102] Wang J., Wakeman T.P., Lathia J.D., Hjelmeland A.B., Wang X.-F., White R.R., Rich J.N., Sullenger B.A. (2009). Notch Promotes Radioresistance of Glioma Stem Cells. Stem Cells.

[103] Lim Y.C., Roberts T.L., Day B.W., Harding A., Kozlov S., Kijas A.W., Ensbey K.S., Walker D.G., Lavin M.F. (2012). A Role for Homologous Recombination and Abnormal Cell-Cycle Progression in Radioresistance of Glioma-Initiating Cells. Mol. Cancer.

[104] Lau J., Ilkhanizadeh S., Wang S., Miroshnikova Y.A., Salvatierra N.A., Wong R.A., Schmidt C., Weaver V.M., Weiss W.A., Persson A.I. (2015). STAT3 Blockade Inhibits Radiation-Induced Malignant Progression in Glioma. Cancer Res..

[105] Li Y., Li A., Glas M., Lal B., Ying M., Sang Y., Xia S., Trageser D., Guerrero-Cázares H., Eberhart C.G. (2011). c-Met signaling induces a reprogramming network and supports the glioblastoma stem-like phenotype. Proc. Natl. Acad. Sci. USA.

[106] Lal B., Xia S., Abounader R., Laterra J. (2005). Targeting the c-Met Pathway Potentiates Glioblastoma Responses to -Radiation. Clin. Cancer Res..

[107] Kim S.-H., Joshi K., Ezhilarasan R., Myers T.R., Siu J., Gu C., Nakano-Okuno M., Taylor D., Minata M., Sulman E.P. (2015). EZH2 Protects Glioma Stem Cells from Radiation-Induced Cell Death in a MELK/FOXM1-Dependent Manner. Stem Cell Rep..

[108] Liu H., Sun Y., Qi X., Gordon R.E., O’Brien J.A., Yuan H., Zhang J., Wang Z., Zhang M., Song Y. (2019). EZH2 Phosphorylation Promotes Self-Renewal of Glioma Stem-Like Cells Through NF-κB Methylation. Front. Oncol..

[109] Wang J., Cheng P., Pavlyukov M.S., Hongyu L., Zhang Z., Kim S.-H., Minata M., Mohyeldin A., Xie W., Chen D. (2017). Targeting NEK2 attenuates glioblastoma growth and radioresistance by destabilizing histone methyltransferase EZH2. J. Clin. Investig..

[110] Jeon H.-Y., Ham S.W., Kim J.-K., Jin X., Lee S.Y., Shin Y.J., Choi C.-Y., Sa J.K., Kim S.H., Chun T. (2019). Ly6G+ inflammatory cells enable the conversion of cancer cells to cancer stem cells in an irradiated glioblastoma model. Cell Death Differ..

[111] Tukachinsky H., Lopez L.V. (2010). A mechanism for vertebrate Hedgehog signaling: Recruitment to cilia and dissociation of SuFu-Gli protein complexes. J. Cell. Biol..

[112] Cheng Y.-C., Shieh S.-Y. (2018). Deubiquitinating enzyme USP3 controls CHK1 chromatin association and activation. Proc. Natl. Acad. Sci. USA.

[113] Tu Y., Chen Z., Zhao P., Sun G., Bao Z., Chao H., Fan L., Li C., You Y., Qu Y. (2020). Smoothened Promotes Glioblastoma Radiation Resistance Via Activating USP3-Mediated Claspin Deubiquitination. Clin. Cancer Res..

[114] Liu T., Huang J. (2016). Replication protein A and more: Single-stranded DNA-binding proteins in eukaryotic cells. Acta Biochim. Et Biophys. Sin..

[115] Pedersen H., Obara E.A.A., Elbæk K.J., Vitting-Serup K., Hamerlik P. (2020). Replication Protein A (RPA) Mediates Radio-Resistance of Glioblastoma Cancer Stem-Like Cells. Int. J. Mol. Sci..

[116] Bartkova B., Hořejší Z., Koed K., Krämer A., Tort F., Zieger K., Guldberg P., Sehested M., Nesland J.M., Lukas C. (2005). DNA damage response as a candidate anti-cancer barrier in early human tumorigenesis. Nat. Cell Biol..

[117] Gaudet S., Branton D., Lue R.A. (2000). Characterization of PDZ-binding kinase, a mitotic kinase. Proc. Natl. Acad. Sci. USA.

[118] Mao P., Bao G., Wang Y.-C., Du C.-W., Yu X., Guo X.-Y., Li R.-C., Wang M.-D. (2020). PDZ-Binding Kinase-Dependent Transcriptional Regulation of CCNB2 Promotes Tumorigenesis and Radio-Resistance in Glioblastoma. Transl. Oncol..

[119] Ichimura K., Pearson D.M., Kocialkowski S., Bäcklund L.M., Chan R., Jones D.T., Collins V.P. (2009). IDH1 mutations are present in the majority of common adult gliomas but rare in primary glioblastomas. Neuro Oncol..

[120] Ceccarelli M., Barthel F.P., Malta T.M., Sabedot T.S., Salama S.R., Murray B.A., Morozova O., Newton Y., Radenbaugh A., Pagnotta S.M. (2016). Molecular Profiling Reveals Biologically Discrete Subsets and Pathways of Progression in Diffuse Glioma. Cell.

[121] Lu C., Ward P.S., Kapoor G.S., Rohle D., Turcan S., Abdel-Wahab O., Edwards C.R., Khanin R., Figueroa M.E., Melnick A. (2012). IDH mutation impairs histone demethylation and results in a block to cell differentiation. Nat. Cell Biol..

[122] Waitkus M.S., Diplas B.H., Yan H. (2016). Isocitrate dehydrogenase mutations in gliomas. Neuro Oncol..

[123] Mellinghoff I.K., Ellingson B.M., Touat M., Maher E., De La Fuente M.I., Holdhoff M., Cote G.M., Burris H., Janku F., Young R.J. (2020). Ivosidenib in Isocitrate Dehydrogenase 1–Mutated Advanced Glioma. J. Clin. Oncol..

[124] Touat M., Idbaih A., Sanson M., Ligon K.L. (2017). Glioblastoma targeted therapy: Updated approaches from recent biological insights. Ann. Oncol..

[125] Weller M., Butowski N., Tran D.D., Recht L.D., Lim M., Hirte H., Ashby L., Mechtler L., A Goldlust S., Iwamoto F. (2017). Rindopepimut with temozolomide for patients with newly diagnosed, EGFRvIII-expressing glioblastoma (ACT IV): A randomised, double-blind, international phase 3 trial. Lancet Oncol..

[126] O’Rourke D.M., Nasrallah M.P., Desai A. (2017). A single dose of peripherally infused EGFRvIII-directed CAR T cells mediates antigen loss and induces adaptive resistance in patients with recurrent glioblastoma. Sci. Transl. Med..

[127] Uhm J.H., Ballman K.V., Wu W., Giannini C., Krauss J., Buckner J.C., James C., Scheithauer B.W., Behrens R.J., Flynn P.J. (2011). Phase II Evaluation of Gefitinib in Patients with Newly Diagnosed Grade 4 Astrocytoma: Mayo/North Central Cancer Treatment Group Study N0074. Int. J. Radiat. Oncol..

[128] Peereboom D.M., Ahluwalia M.S., Ye X., Supko J.G., Hilderbrand S.L., Phuphanich S., Nabors L.B., Rosenfeld M.R., Mikkelsen T., Grossman S.A. (2013). NABTT 0502: A phase II and pharmacokinetic study of erlotinib and sorafenib for patients with progressive or recurrent glioblastoma multiforme. Neuro Oncol..

[129] AbbVie Provides Update on Depatuxizumab Mafodotin (Depatux-M), An Investigational Medicine for Newly Diagnosed Glioblastoma, an AGGRESSIVE Form of Brain Cancer. https://news.abbvie.com/news/press-releases/abbvie-provides-update-on-depatuxizumab-mafodotin-depatux-m-an-investigational-medicine-for-newly-diagnosed-glioblastoma-an-aggressive-form-brain-cancer.htm.

[130] Hasselbalch B., Lassen U., Hansen S., Holmberg M., Sørensen M., Kosteljanetz M., Broholm H., Stockhausen M.-T., Poulsen H.S. (2010). Cetuximab, bevacizumab, and irinotecan for patients with primary glioblastoma and progression after radiation therapy and temozolomide: A phase II trial. Neuro Oncol..

[131] Klingler S., Guo B., Yao J., Yan H., Zhang L., Vaseva A.V., Chen S., Canoll P., Horner J.W., Wang Y.A. (2015). Development of Resistance to EGFR-Targeted Therapy in Malignant Glioma Can Occur through EGFR-Dependent and -Independent Mechanisms. Cancer Res..

[132] Lassman A.B., Pugh S.L., Gilbert M.R., Aldape K.D., Geinoz S., Beumer J.H., Christner S.M., Komaki R., DeAngelis L.M., Gaur R. (2015). Phase 2 trial of dasatinib in target-selected patients with recurrent glioblastoma (RTOG 0627). Neuro Oncol..

[133] di Stefano A.L., Fucci A., Frattini V., Labussiere M., Mokhtari K., Zoppoli P., Marie Y., Bruno A., Boisselier B., Savatovsky M.G.J. (2015). Detection, Characterization, and Inhibition of FGFR-TACC Fusions in IDH Wild-type Glioma. Clin. Cancer Res..

[134] Chi A.S., Batchelor T.T., Kwak E.L., Clark J.W., Wang D.L., Wilner K.D., Louis D.N., Iafrate A.J. (2012). Rapid Radiographic and Clinical Improvement After Treatment of a MET-Amplified Recurrent Glioblastoma with a Mesenchymal-Epithelial Transition Inhibitor. J. Clin. Oncol..

[135] Ma D.J., Galanis E., Anderson S.K., Schiff D., Kaufmann T.J., Peller P.J., Giannini C., Brown P.D., Uhm J.H., McGraw S. (2015). A phase II trial of everolimus, temozolomide, and radiotherapy in patients with newly diagnosed glioblastoma: NCCTG N057K. Neuro Oncol..

[136] Lassen U., Mau-Sørensen M., Gaziel T.B., Hasselbalch B., Poulsen H.S. (2013). Phase II study of bevacizumab and temsirolimus combination therapy for recurrent glioblastoma multiforme. Anticancer. Res..

[137] Schreck K.C., A Grossman S., Pratilas C. (2019). BRAF Mutations and the Utility of RAF and MEK Inhibitors in Primary Brain Tumors. Cancers.

[138] Hottinger A.F., Ben Aissa A., Espeli V., Squiban D., Dunkel N., I Vargas M., Hundsberger T., Mach N., Schaller K., Weber D.C. (2014). Phase I study of sorafenib combined with radiation therapy and temozolomide as first-line treatment of high-grade glioma. Br. J. Cancer.

[139] Costa B., Bendinelli S., Gabelloni P., Da Pozzo E., Daniele S., Scatena F., Vanacore R., Campiglia P., Bertamino A., Gomez-Monterrey I. (2013). Human Glioblastoma Multiforme: p53 Reactivation by a Novel MDM2 Inhibitor. PLoS ONE.

[140] Verreault M., Schmitt C., Goldwirt L., Pelton K., Haidar S., Levasseur C., Guehennec J., Knoff D.S., Labussière M., Marie Y. (2016). Preclinical Efficacy of the MDM2 Inhibitor RG7112 in MDM2-Amplified and TP53 Wild-type Glioblastomas. Clin. Cancer Res..

[141] Patnaik A., Rosen L.S., Tolaney S.M., Tolcher A.W., Goldman J.W., Gandhi L., Papadopoulos K.P., Beeram M., Rasco D.W., Hilton J.F. (2016). Efficacy and Safety of Abemaciclib, an Inhibitor of CDK4 and CDK6, for Patients with Breast Cancer, Non-Small Cell Lung Cancer, and Other Solid Tumors. Cancer Discov..

[142] Tien A.-C., Li J., Bao X., Derogatis A., Kim S., Mehta S., Sanai N. (2019). A Phase 0 Trial of Ribociclib in Recurrent Glioblastoma Patients Incorporating a Tumor Pharmacodynamic- and Pharmacokinetic-Guided Expansion Cohort. Clin. Cancer Res..

[143] Nguyen L.V., Searle K., Jerzak K.J. (2019). Central nervous system-specific efficacy of CDK4/6 inhibitors in randomized controlled trials for metastatic breast cancer. Oncotarget.

[144] Michaud K., Solomon D.A., Oermann E., Kim J.-S., Zhong W.-Z., Prados M.D., Ozawa T., James C.D., Waldman T. (2010). Pharmacologic Inhibition of Cyclin-Dependent Kinases 4 and 6 Arrests the Growth of Glioblastoma Multiforme Intracranial Xenografts. Cancer Res..

[145] Gilbert M.R., Dignam J.J., Armstrong T.S., Wefel J.S., Blumenthal D.T., Vogelbaum M.A., Colman H., Chakravarti A., Pugh S., Won M. (2014). A Randomized Trial of Bevacizumab for Newly Diagnosed Glioblastoma. N. Engl. J. Med..

[146] Brahmer J., Reckamp K.L., Baas P., Crinò L., Eberhardt W.E.E., Poddubskaya E., Antonia S., Pluzanski A., Vokes E.E., Holgado E. (2015). Nivolumab versus Docetaxel in Advanced Squamous-Cell Non–Small-Cell Lung Cancer. N. Engl. J. Med..

[147] Robert C., Long G.V., Brady B. (2015). Nivolumab in previously untreated melanoma without BRAF mutation. N. Engl. J. Med..

[148] Liau L.M., Ashkan K., Tran D.D. (2018). First results on survival from a large Phase 3 clinical trial of an autologous dendritic cell vaccine in newly diagnosed glioblastoma. J. Transl. Med..

[149] Reardon D.A., Omuro A., Brandes A.A., Rieger J., Wick A., Sepulveda J., Phuphanich S., De Souza P., Ahluwalia M.S., Lim M. (2017). OS10.3 Randomized Phase 3 Study Evaluating the Efficacy and Safety of Nivolumab vs Bevacizumab in Patients With Recurrent Glioblastoma: CheckMate 143. Neuro Oncol..

[150] Chongsathidkiet P., Jackson C., Koyama S. (2019). Sequestration of T cells in bone marrow in the setting of glioblastoma and other intracranial tumors [published correction appears in Nat Med]. Nat. Med..

[151] Dieckmann D., Plottner H., Berchtold S., Berger T., Schuler G. (2001). Ex Vivo Isolation and Characterization of Cd4+Cd25+ T Cells with Regulatory Properties from Human Blood. J. Exp. Med..

[152] Fecci P.E., Mithcell D.A., Whitesides J.F., Xie W., Friedman A.H., Archer G.E., Herndon J.E., Bigner D.D., Dranoff G., Sampson J.H. (2006). Increased regulatory T-cell fraction amidst a diminished CD4 compartment explains cellular immune defects in patients with malignant glioma. Cancer Res..

[153] Andaloussi A.E., Lesniak M.S. (2006). An increase in CD4+CD25+FOXP3+ regulatory T cells in tumor-infiltrating lymphocytes of human glioblastoma multiforme. Neuro Oncol..

[154] Strand S., Hofmann W.J., Hug H. (1996). Lymphocyte apoptosis induced by CD95 (APO-1/Fas) ligand-expressing tumor cells—a mechanism of immune evasion?. Nat. Med..

[155] Woroniecka K., Chongsathidkiet P., Rhodin K., Kemeny H., DeChant C., Farber S.H., Elsamadicy A.A., Cui X., Koyama S., Jackson C. (2018). T-Cell Exhaustion Signatures Vary with Tumor Type and Are Severe in Glioblastoma. Clin. Cancer Res..

[156] Camisaschi C., Casati C., Rini F., Perego M., De Filippo A., Triebel F., Parmiani G., Belli F., Rivoltini L., Castelli C. (2010). LAG-3 Expression Defines a Subset of CD4+CD25highFoxp3+ Regulatory T Cells That Are Expanded at Tumor Sites. J. Immunol..

[157] Baeza N., Weller M., Yonekawa Y., Kleihues P., Ohgaki H. (2003). PTEN methylation and expression in glioblastomas. Acta Neuropathol..

[158] Waldron J.S., Yang I., Han S., Tihan T., Sughrue M.E., Mills S.A., Pieper R.O., Parsa A.T. (2010). Implications for immunotherapy of tumor-mediated T-cell apoptosis associated with loss of the tumor suppressor PTEN in glioblastoma. J. Clin. Neurosci..

[159] Peng W., Chen J.Q., Liu C., Malu S., Creasy C., Tetzlaff M.T., Xu C., McKenzie J.A., Zhang C., Liang X. (2015). Loss of PTEN Promotes Resistance to T Cell-Mediated Immunotherapy. Cancer Discov..

[160] Chen Q., Han B., Meng X., Duan C., Yang C., Wu Z., Magafurov D., Zhao S., Safin S., Jiang C. (2019). Immunogenomic analysis reveals LGALS1 contributes to the immune heterogeneity and immunosuppression in glioma. Int. J. Cancer.

[161] Hahn S.A., Stahl H.F., Becker C., Correll A., Schneider F.-J., Tuettenberg A., Jonuleit H. (2013). Soluble GARP has potent antiinflammatory and immunomodulatory impact on human CD4+ T cells. Blood.

[162] Zimmer N., Kim E.L., Sprang B., Leukel P., Khafaji F., Ringel F., Sommer C.J., Tuettenberg A. (2019). GARP as an Immune Regulatory Molecule in the Tumor Microenvironment of Glioblastoma Multiforme. Int. J. Mol. Sci..

[163] Wu A., Wei J., Kong L.-Y., Wang Y., Priebe W., Qiao W., Sawaya R., Heimberger A.B. (2010). Glioma cancer stem cells induce immunosuppressive macrophages/microglia. Neuro Oncol..

[164] Hussain S.F., Yang D., Suki D., Aldape K., Grimm E., Heimberger A.B. (2006). The role of human glioma-infiltrating microglia/macrophages in mediating antitumor immune responses1. Neuro Oncol..

[165] Kryczek I., Wei S., Zou L., Altuwaijri S., Szeliga W., Kolls J., Chang A., Zou W. (2007). Cutting edge: Th17 and regulatory T cell dynamics and the regulation of IL-2 in the tumor microenvironment. J. Immunol..

[166] Hussain S.F., Kong L.-Y., Jordan J., Conrad C., Madden T., Fokt I., Priebe W., Heimberger A.B. (2007). A Novel Small Molecule Inhibitor of Signal Transducers and Activators of Transcription 3 Reverses Immune Tolerance in Malignant Glioma Patients. Cancer Res..

[167] Kortylewski M., Kujawski M., Wang T., Wei S., Zhang S., Pilon-Thomas S., Niu G., Kay H., Mule J.J., Kerr W.G. (2005). Inhibiting Stat3 signaling in the hematopoietic system elicits multicomponent antitumor immunity. Nat. Med..

[168] Akiyama Y., Nonomura C., Ashizawa T., Iizuka A., Kondou R., Miyata H., Sugino T., Mitsuya K., Hayashi N., Nakasu Y. (2017). The anti-tumor activity of the STAT3 inhibitor STX-0119 occurs via promotion of tumor-infiltrating lymphocyte accumulation in temozolomide-resistant glioblastoma cell line. Immunol. Lett..

[169] Alvarado A.G., Thiagarajan P.S., Mulkearns-Hubert E.E., Silver D.J., Hale J.S., Alban T.J., Turaga S.M., Jarrar A., Reizes O., Longworth M.S. (2017). Glioblastoma Cancer Stem Cells Evade Innate Immune Suppression of Self-Renewal through Reduced TLR4 Expression. Cell Stem Cell.

[170] Almand B., Clark J.I., Nikitina E., Van Beynen J., English N.R., Knight S.C., Carbone D.P., Gabrilovich D.I. (2001). Increased Production of Immature Myeloid Cells in Cancer Patients: A Mechanism of Immunosuppression in Cancer. J. Immunol..

[171] Gieryng A., Pszczolkowska D., A Walentynowicz K., Rajan W.D., Kaminska B. (2017). Immune microenvironment of gliomas. Lab. Investig..

[172] Marigo I., Bosio E., Solito S., Mesa C., Fernandez A., Dolcetti L. (2010). Tumor-induced tolerance and immune suppression depend on the C/EBPbeta transcription factor. Immunity.

[173] Bah I., Kumbhare A., Nguyen L., McCall C.E., El Gazzar M. (2018). IL-10 induces an immune repressor pathway in sepsis by promoting S100A9 nuclear localization and MDSC development. Cell. Immunol..

[174] Lechner M.G., Megiel C., Russell S.M., Bingham B., Arger N., Woo T., Epstein A.L. (2011). Functional characterization of human Cd33+ And Cd11b+ myeloid-derived suppressor cell subsets induced from peripheral blood mononuclear cells co-cultured with a diverse set of human tumor cell lines. J. Transl. Med..

[175] Nagaraj S., Schrum A.G., Cho H.-I., Celis E., Gabrilovich D.I. (2010). Mechanism of T Cell Tolerance Induced by Myeloid-Derived Suppressor Cells. J. Immunol..

[176] Park M.-J., Lee S.-H., Kim E.-K., Lee E.-J., Baek J.-A., Park S.-H., Kwok S.-K., Cho M.-L. (2018). Interleukin-10 produced by myeloid-derived suppressor cells is critical for the induction of Tregs and attenuation of rheumatoid inflammation in mice. Sci. Rep..

[177] Ostrand-Rosenberg S., Sinha P., Beury D.W., Clements V.K. (2012). Cross-talk between myeloid-derived suppressor cells (MDSC), macrophages, and dendritic cells enhances tumor-induced immune suppression. Semin. Cancer Biol..

[178] Comba A., Dunn P.J., E Argento A., Kadiyala P., Ventosa M., Patel P., Zamler D.B., Núñez F.J., Zhao L., Castro M.G. (2020). Fyn tyrosine kinase, a downstream target of receptor tyrosine kinases, modulates antiglioma immune responses. Neuro Oncol..

[179] Topalian S.L., Hodi F.S., Brahmer J.R., Gettinger S.N., Smith D.C., McDermott D.F., Powderly J.D., Carvajal R.D., Sosman J.A., Atkins M.B. (2012). Safety, Activity, and Immune Correlates of Anti–PD-1 Antibody in Cancer. N. Engl. J. Med..

[180] Rizvi N.A., Hellmann M.D., Snyder A., Kvistborg P., Makarov V., Havel J.J., Lee W., Yuan J., Wong P., Ho T.S. (2015). Mutational landscape determines sensitivity to PD-1 blockade in non–small cell lung cancer. Science.

[181] Hodges T.R., Ott M., Xiu J., Gatalica Z., Swensen J., Zhou S., Huse J.T., De Groot J., Li S., Overwijk W.W. (2017). Mutational burden, immune checkpoint expression, and mismatch repair in glioma: Implications for immune checkpoint immunotherapy. Neuro Oncol..

[182] McGranahan N., Furness A.J.S., Rosenthal R., Ramskov S., Lyngaa R.B., Saini S.K., Jamal-Hanjani M., Wilson G.A., Birkbak N.J., Hiley C.T. (2016). Clonal neoantigens elicit T cell immunoreactivity and sensitivity to immune checkpoint blockade. Science.

[183] Zhao J., Chen A.X., Gartrell R.D., Silverman A.M., Aparicio L., Chu T., Bordbar D., Shan D., Samanamud J., Mahajan A. (2019). Immune and genomic correlates of response to anti-PD-1 immunotherapy in glioblastoma. Nat. Med..

[184] Schalper K.A., Rodriguez-Ruiz M.E., Diez-Valle R., López-Janeiro A., Porciuncula A., Idoate M.A., Inogés S., De Andrea C., De Cerio A.L.-D., Tejada S. (2019). Neoadjuvant nivolumab modifies the tumor immune microenvironment in resectable glioblastoma. Nat. Med..

[185] Giles A.J., Hutchinson M.-K.N.D., Sonnemann H.M., Jung J., E Fecci P., Ratnam N.M., Zhang W., Song H., Bailey R., Davis D. (2018). Dexamethasone-induced immunosuppression: Mechanisms and implications for immunotherapy. J. Immunother. Cancer.

[186] Mathios D., Kim J.E., Mangraviti A., Phallen J., Park C.-K., Jackson C.M., Garzon-Muvdi T., Kim E., Theodros D., Polanczyk M. (2016). Anti-PD-1 antitumor immunity is enhanced by local and abrogated by systemic chemotherapy in GBM. Sci. Transl. Med..

[187] Dutoit V., Philippin G., Widmer V., Marinari E., Vuilleumier A., Migliorini D., Schaller K., Dietrich P.-Y. (2020). Impact of Radiochemotherapy on Immune Cell Subtypes in High-Grade Glioma Patients. Front. Oncol..

[188] Miyazaki T., Ishikawa E., Matsuda M., Sugii N., Kohzuki H., Akutsu H., Sakamoto N., Takano S., Matsumura A. (2020). Infiltration of CD163-positive macrophages in glioma tissues after treatment with anti-PD-L1 antibody and role of PI3Kγ inhibitor as a combination therapy with anti-PD-L1 antibody in in vivo model using temozolomide-resistant murine glioma-initiating cells. Brain Tumor Pathol..

[189] Brown C.E., Alizadeh D., Starr R., Weng L., Wagner J.R., Naranjo A., Ostberg J.R., Blanchard M.S., Kilpatrick J., Simpson J. (2016). Regression of Glioblastoma after Chimeric Antigen Receptor T-Cell Therapy. New Engl. J. Med..

[190] Keskin D.B., Anandappa A.J., Sun J., Mints M., Mathewson N.D., Li S., Oliveira G., Giobbie-Hurder A., Felt K., Gjini E. (2018). Neoantigen vaccine generates intratumoral T cell responses in phase Ib glioblastoma trial. Nature.

[191] Rampling R., Peoples S., Mulholland P.J., James A., Al-Salihi O., Twelves C.J., McBain C., Jefferies S., Jackson A., Wa R.J. (2016). A Cancer Research UK First Time in Human Phase I Trial of IMA950 (Novel Multipeptide Therapeutic Vaccine) in Patients with Newly Diagnosed Glioblastoma. Clin. Cancer Res..

[192] Wen P.Y., Reardon D.A., Armstrong T.S., Phuphanich S., Aiken R.D., Landolfi J.C., Curry W.T., Zhu J., Glantz M., Peereboom D.M. (2005). A randomized, double-blind, placebo-controlled phase 2 trial of dendritic cell (DC) vaccination with ICT-107 in newly diagnosed glioblastoma (GBM) patients. J. Clin. Oncol..

[193] Lang F.F., Conrad C., Gomez-Manzano C., Yung W.A., Sawaya R., Weinberg J.S., Prabhu S.S., Rao G., Fuller G.N., Aldape K.D. (2018). Phase I Study of DNX-2401 (Delta-24-RGD) Oncolytic Adenovirus: Replication and Immunotherapeutic Effects in Recurrent Malignant Glioma. J. Clin. Oncol..

[194] Van den Bossche W.B., Kleijn A., Teunissen C.E., Voerman J.S., Teodosio C., Noske D.P., van Dongen J.J.M., Dirven C.M.F., Lamfers M.L.M. (2018). Oncolytic virotherapy in glioblastoma patients induces a tumor macrophage phenotypic shift leading to an altered glioblastoma microenvironment. Neuro Oncol..

[195] Brown M.C., Holl E.K., Boczkowski D., Dobrikova E., Mosaheb M., Chandramohan V., Bigner D.D., Gromeier M., Nair S.K. (2017). Cancer immunotherapy with recombinant poliovirus induces IFN-dominant activation of dendritic cells and tumor antigen–specific CTLs. Sci. Transl. Med..

[196] Hardcastle J., Mills L., Malo C.S., Jin F., Kurokawa C., Geekiyanage H., Schroeder M., Sarkaria J., Johnson A.J., Galanis E. (2016). Immunovirotherapy with measles virus strains in combination with anti–PD-1 antibody blockade enhances antitumor activity in glioblastoma treatment. Neuro Oncol..

[197] Wu A., Maxwell R., Xia Y., Cardarelli P., Oyasu M., Belcaid Z., Kim E., Hung A., Luksik A.S., Garzon-Muvdi T. (2019). Combination anti-CXCR4 and anti-PD-1 immunotherapy provides survival benefit in glioblastoma through immune cell modulation of tumor microenvironment. J. Neuro Oncol..

